# Irradiation induces p53 loss of heterozygosity in breast cancer expressing mutant p53

**DOI:** 10.1038/s42003-019-0669-y

**Published:** 2019-11-27

**Authors:** Amr Ghaleb, Alisha Yallowitz, Natalia Marchenko

**Affiliations:** 1 Department of Pathology, Stony Brook University, Stony Brook, NY 11794-8691 USA; 2 Weill Cornell Medicine, 1300 York Avenue, LC-902, New York, NY 10065 USA

**Keywords:** Breast cancer, Cancer, Cell-cycle exit, Checkpoints, Molecular biology

## Abstract

Mutations in one allele of the TP53 gene in cancer early stages are frequently followed by the loss of the remaining wild-type allele (LOH) during tumor progression. However, the clinical impact of TP53 mutations and p53LOH, especially in the context of genotoxic modalities, remains unclear. Using MMTV;ErbB2 model carrying a heterozygous R172H p53 mutation, we report a previously unidentified oncogenic activity of mutant p53 (mutp53): the exacerbation of p53LOH after irradiation. We show that wild-type p53 allele is partially transcriptionally competent and enables the maintenance of the genomic integrity under normal conditions in mutp53 heterozygous cells. In heterozygous cells γ-irradiation promotes mutp53 stabilization, which suppresses DNA repair and the cell cycle checkpoint allowing cell cycle progression in the presence of inefficiently repaired DNA, consequently increases genomic instability leading to p53LOH. Hence, in mutp53 heterozygous cells, irradiation facilitates the selective pressure for p53LOH that enhances cancer cell fitness and provides the genetic plasticity for acquiring metastatic properties.

## Abstract (web summary)

Amr Ghaleb et al. find that, in an ErbB2 mouse model, irradiation of premalignant mammary lesions enhances tumorigenesis in tumours that are heterozygous for p53 containing a p53 R172H mutation. This is associated with loss of the wild-type p53 allele, stabilisation of mutant p53 protein and activation of mTOR signaling.

## Introduction

Breast cancer is one of the leading causes of death in women worldwide. Currently, radiation therapy, coupled with breast-conserving surgery is the standard of care for the majority of breast cancer patients. However, a meta-analysis showed that radiation reduces 15-year breast cancer mortality risk only by 5%^1^. At present, 30% of all breast cancer cases are considered to be overtreated by the administration of more aggressive therapies than is necessary or by overdiagnosis, where no treatment is required^2^. An estimated one to three deaths from overtreatment occur for every one breast cancer death avoided^3^. Hence, the understanding of how to reliably identify which breast cancer patients will benefit from radiotherapy is needed to reduce the mortality risk and improve the quality of life.

Mutations in TP53 (p53) gene are common in breast cancer and are especially enriched in Her2 (human EGF receptor 2, ErbB2) positive breast cancer (72%)^4^, and basal-like breast cancer (80%)^4^. Whereas wild-type p53 (wtp53) is an important determinant of the efficacy of DNA-damaging therapies, the p53 mutational status is not routinely used for cancer management. This is mainly due to inconsistent results of clinical studies^5^, conceivably because in previous studies the predictive effect of p53 status in response to genotoxic modalities has not been assessed at the different stages and in the context of p53 heterozygosity. In some cancers, mutant p53 (mutp53) status was shown to predict poor patient outcome in response to genotoxic treatment^6^, whereas other studies showed a better response of mutp53 tumors to chemotherapies^7^. Thus, knowing how mutp53 interacts with the specific oncogenic environment in the context of conventional therapies will facilitate the clinical utilization of the mutational status of p53.

Clinical data suggest that p53 behaves as a classic “two-hit” tumor suppressor where a point mutation in one allele of p53 at early stages is followed by loss of the wild-type allele (loss of heterozygosity (LOH)) later during tumor progression^8^. Albeit mutp53 in heterozygosity may exert dominant-negative (DN) effect^9^, several *in vivo* studies showed that wtp53 retains its function in heterozygous tumors^10^. In support, ~80% of advanced-stage mutp53 breast cancer tumors have lost the wtp53 allele suggesting the high selective pressure for p53LOH during tumor progression^11^. These studies raise the question of why mutp53 exerts DN in some contexts, but not others, and what is the clinical relevance of these findings?

To address these questions, we generate MMTV-ErbB2 and mutp53 R172H (H thereafter) knock-in mouse model that faithfully recapitulates human Her2-positive breast cancer^12^. We find that wtp53 retains its transcriptional activity in both p53−/+;ErbB2 and p53H/+;ErbB2 heterozygous cancer cells. However, irradiation of pre-malignant mammary lesions aggravates mammary tumorigenesis that is associated with increased frequency of p53LOH mostly in mutp53 heterozygous mice. Importantly, p53LOH is concomitant with elevated genomic and chromosomal aberrations, inefficient DNA repair, activation of mTOR signaling and, as a result, increased metastases in mutp53 heterozygous compared to hemizygous cells. Hence, we propose that in response to irradiation, mutp53, via activation of the mTOR pathway, generates the selective pressure for wtp53 loss in heterozygous cells that is fueled by deficient DNA repair and abnormal cell-cycle progression.

## Results

### Survival of mutp53 breast cancer patients following radiotherapy is stage-dependent

**Fig. 1:**
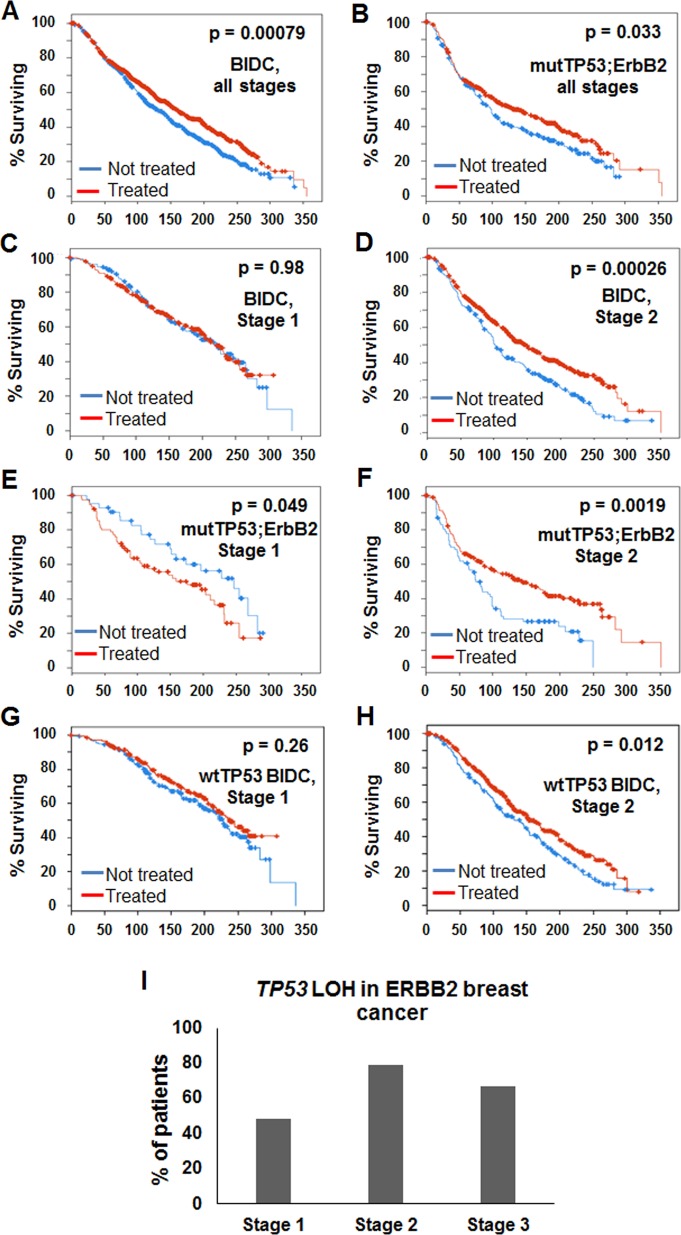
The survival of mutp53 breast cancer patients following radiotherapy is stage-dependent. Kaplan-Meier survival curve for patients (*n* = 2433) with breast cancer receiving radiation therapy (red line) or untreated (blue line). **a** All breast cancer patients (*p* = 0.00079). **b** Patients with TP53 and ErbB2 mutations (*n* = 193, *p* = 0.033). **c** Stage 1, all breast cancer patients (*n* = 440, *p* = 0.98). **d** Stage 2, all breast cancer patients (*n* = 759, *p* = 0.00026). **e** Stage 1 patients with TP53 and ErbB2 mutations (*n* = 35, *p* = 0.049). **f** Stage 2 patients with TP53 and ErbB2 mutations (*n* = 70, *p* = 0.0019). **g** Stage 1 patients with wild-type p53 (*n* = 302, *p* = 0.26). **h** Stage 2 patients with wild-type p53 (*n* = 458, *p* = 0.017). **i** TP53 LOH in breast cancer patients is stage-dependent. 52% of stage I and only 20% stage II mutp53 tumors retain wtp53 allele.

To assess the predictive effect of TP53 overall mutations in response to γ-irradiation, we investigated publicly available databases of retrospective clinical data of Metabric cohort (2433 breast cancer patients, http://www.cbioportal.org). Analysis of all stages of breast invasive ductal carcinoma (BIDC) combined, showed that radiotherapy improved overall survival (OS) to all patients independently of the mutational profile (Fig. 1a, b). Stratification of BIDC patients by stage demonstrated a significant stage-dependent benefit of radiotherapy in stage 2 compared to stage 1 cohort (Fig. 1c, d). Strikingly, stratification by p53 status in ErbB2 cohort of BIDC showed that stage 1 patients with overall mutant TP53;ErbB2 tumors had significantly worse OS after radiotherapy as compared to untreated cohorts (Fig. 1e), while radiation significantly improved OS of stage 2 patients with overall mutant TP53;ErbB2 tumors (Fig. 1f). In contrast, radiotherapy marginally extended OS of patients with wild-type TP53 tumors independently of the stage (Fig. 1g, h). Hence, in ErbB2 breast cancer patients, overall mutant TP53 status might be predictive of a negative outcome from genotoxic modalities in stage 1, whereas it is significantly advantageous for stage 2. Similarly, patients with overall mutant TP53 BIDC tumors showed a worse outcome after chemotherapy in stage 1 (Supplementary Fig. 1a), but favorable outcome in stage 2 (Supplementary Fig. 1b). Although the number of cases limited the statistical significance, this trend was not observed for patients with wild-type TP53 breast cancer patients after chemotherapy (Supplementary Fig. 1c, d). Importantly, the frequency of p53LOH increased during tumor progression: 52% of stage 1 patients are heterozygous for p53, while only 20% of stage 2 patients retain wtp53 allele (Fig. 1i).

Therefore, we hypothesized that the p53LOH status might be an important determinant of the survival of patients carrying mutant TP53 tumors after genotoxic therapies.

### γ-Irradiation aggravates mammary tumorigenesis and promotes p53LOH in MMTV-ErbB2 mouse model

**Fig. 2:**
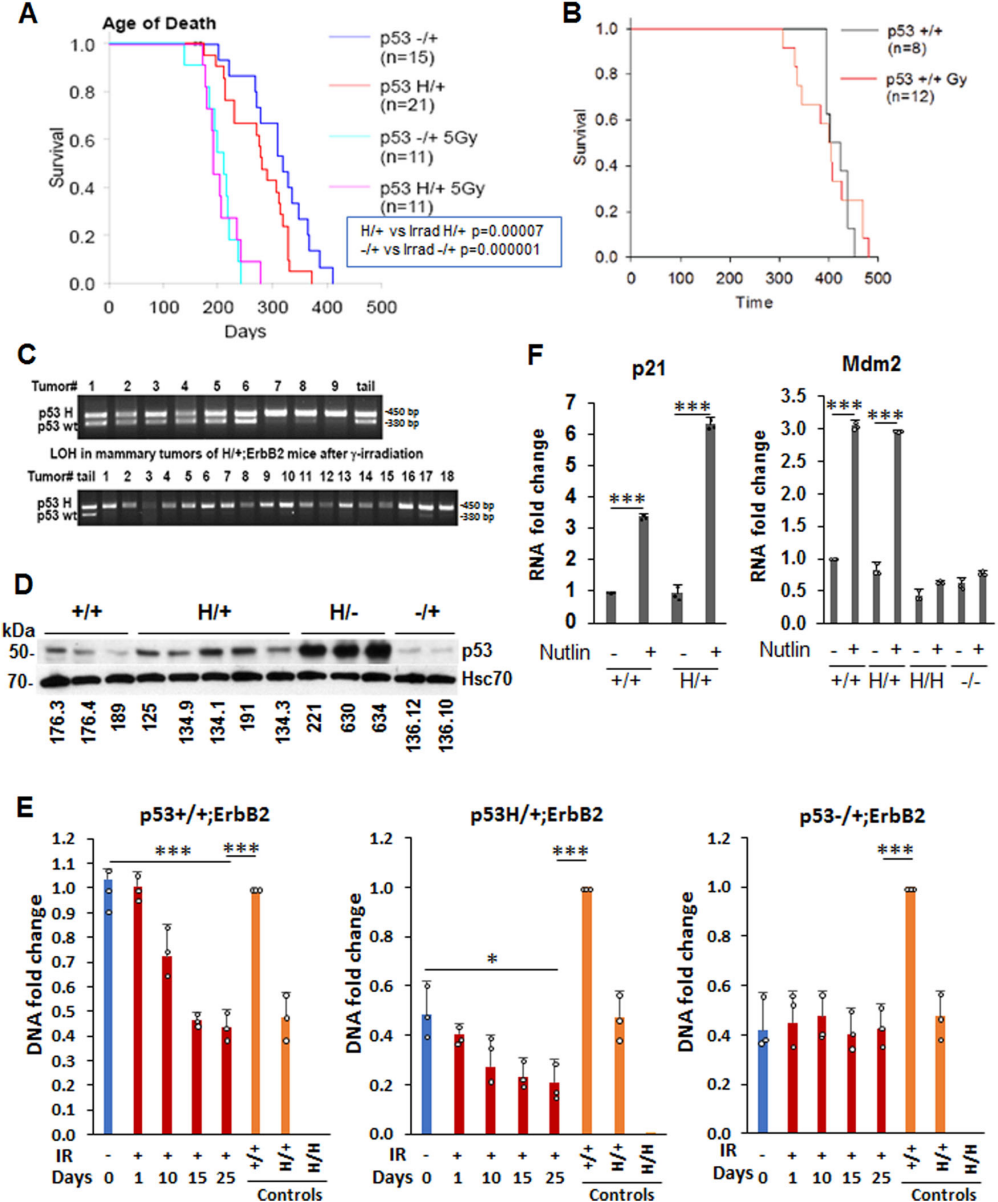
γ-Irradiation aggravates mammary tumorigenesis and promotes p53LOH in MMTV-ErbB2 mouse model. **a**, **b** Kaplan-Meier survival curves of irradiated and non-irradiated MMTV-ErbB2 mouse model. Single-dose of 5 Gy γ-irradiation at the time of onset of pre-malignant lesions (80 days) aggravates mammary tumorigenesis in p53H/+;ErbB2 vs. p53−/+;ErbB2 (*p* < 0.001 and *p* = 0.04, respectively) (**a**), but not in p53+/+;ErbB2 mice (*p* = 0.892) (**b**). n values are indicated in the figure and represent the number of mice. **c** Example of LOH analysis in tumors from p53H/+;ErbB2 mice. Non-irradiated mice are showing LOH in few mice only (top lanes 7–9). Irradiated mice, showing LOH in all but 1 mouse (bottom lane 17). **d** P53 expression in a panel of cell lines established from mammary tumors of MMTV-ErbB2 mice with different p53 genotypes. HSC70 is a loading control. **e** Mutp53 enhances LOH following γ-irradiation in cell culture (*n* = 3 independent samples Error bars represent ± SD). Cultivated mammary tumors cells were irradiated (9 Gy), or not, and grown up to 25 days post-irradiation. DNA was extracted at the indicated time points. The copy number of p53 wt and mut alleles was quantified by real-time PCR. DNA extracted from tail tissue samples of the corresponding genotype was used for copy number control. The experiment was repeated three times. Summary of a representative experiment. **f** Wtp53 retains transcriptional activity and, in response to Mdm2 inhibitor nutlin, induces its target p21 and Mdm2 in mutp53 heterozygous cells. Nutlin does not induce Mdm2 in p53H/H;ErbB2 and p53−/−;ErbB2 MECs. *n* = 3 independent experiments. Error bars represent ± SD.

To recapitulate the early stages of human ErbB2 breast cancer and study the impact of p53LOH in context of genotoxic therapies, we generated a genetic mouse model as described before^12^. The murine R172H (H thereafter) p53 mutation corresponds to human hotspot R175H mutation in ErbB2 breast cancer^4^ and (http://www.cbioportal.org)). We found that ErbB2 mammary tumorigenesis was aggravated in p53H/+;ErbB2 mice, compared to p53 null counterparts, indicated by earlier tumor onset and shorter survival (Fig. 2, Table 1,^12^). Furthermore, a single dose of γ-irradiation at the time of pre-malignant lesions onset (5 Gy to 80-day-old mice) significantly shortened both tumor latency and overall survival by approximately 80 days, in both p53−/+;ErbB2 and p53H/+;ErbB2 genotypes (Fig. 2a), but not in p53+/+;ErbB2 mice (Fig. 2b). Notably, irradiation increased p53LOH in both p53H/+;ErbB2 (Fig. 2c) and p53−/+;ErbB2 (Table 1) tumors. Also, p53LOH occurred more frequently in the presence of mutp53 allele in both non-irradiated p53H/+;ErbB2 and p53−/+;ErbB2 (18 and 11%, respectively, Table 1) and this difference was exacerbated after irradiation (p53H/+;ErbB2 95% vs. p53−/+;ErbB2 38%, respectively, Table 1). Although we did not observe survival difference in p53H/+;ErbB2 vs. p53−/+;ErbB2, the main phenotype associated with enhanced p53LOH was the increased rate of metastases in the presence of mutp53 allele (p53H/+;ErbB2 100% vs. p53+/−;ErbB2 58%, Table 1). Contrary, irradiation of p53+/+;ErbB2 mice did not increase metastasis, and we were unable to detect loss of wtp53 allele in tumors after irradiation of p53+/+;ErbB2 mice (Table 1).

**Table 1:** Median survival, tumor spectrum and LOH in mice with indicated genotypes.

Tumor types				Irradiated		
	−/+;ErbB2 (*n* = 19)	H/+;ErbB2 (*n* = 22)	+/+;ErbB2 (*n* = 9)	−/+;ErbB2 (*n* = 16)	H/+;ErbB2 (*n* = 17)	+/+;ErbB2 (*n* = 16)
Mammary	86%	95%	100%	75%	59%	88%
Lymphoma	10%	0%	0	19%	24%	6%
Mammary + Lymphoma	0%	0	0	6%	0%	0%
Sarcoma	4%	5%	0	0%	24%	6%
tumor number per mouse	2.7	6.1	4.8	3.6	5.1	2.6
Median survival (days)	312	285	399	2.3	2.5	397
% of lung metastases	52%	58%	50%	58%	100%	54%
loss of wtp53 allele	11%	18%	0%	38%	95%	0%

Next, we determined whether the presence of mutp53 allele accelerates p53LOH after irradiation in vitro. Cell lines, generated from mouse tumors of different genotypes (Fig. 2d and Supplementary Fig. 2a) were irradiated, or not, and the copy number of wtp53 and mutp53 alleles were determined at different time points by qPCR (Fig. 2e). In agreement with *in vivo* data (Table 1), we found 3-fold reduction of wtp53 allele post-irradiation in p53H/+;ErbB2 compared to untreated cells (a 5-fold reduction compared to control p53+/+;ErbB2 cells), but not in p53−/+;ErbB2 cells compared to non-irradiated cells (Fig. 2e). Irradiation induced a 2-fold decrease in copy number of the wild-type allele in p53+/+;ErbB2 cells compared to control cells (Fig. 2e).

To evaluate the consequences of p53LOH in vitro with respect to the transcriptional activity of wtp53 in heterozygosity, we examined the expression of canonical p53 target genes Mdm2 and p21 in response to Mdm2 inhibitor, nutlin, by qPCR. Nutlin promotes p53 transcriptional activity without induction of DNA damage^13^. No significant difference in the expression of Mdm2 and p21 was observed between p53+/+;ErbB2 and p53H/+;ErbB2 cells at basal level, while the expression of both was increased following nutlin addition (Fig. 2f). In contrast, nutlin failed to induce p53 targets in p53H/H;ErbB2 and p53−/−;ErbB2 mammary epithelial cells (MECs) (Fig. 2f). Hence, in heterozygosity, wtp53 at least partially preserves its transcriptional function, while p53LOH may abrogate tumor-suppressor activities of wtp53.

### Irradiation induces the accumulation of mutant p53 protein in heterozygous cancer cells

**Fig. 3:**
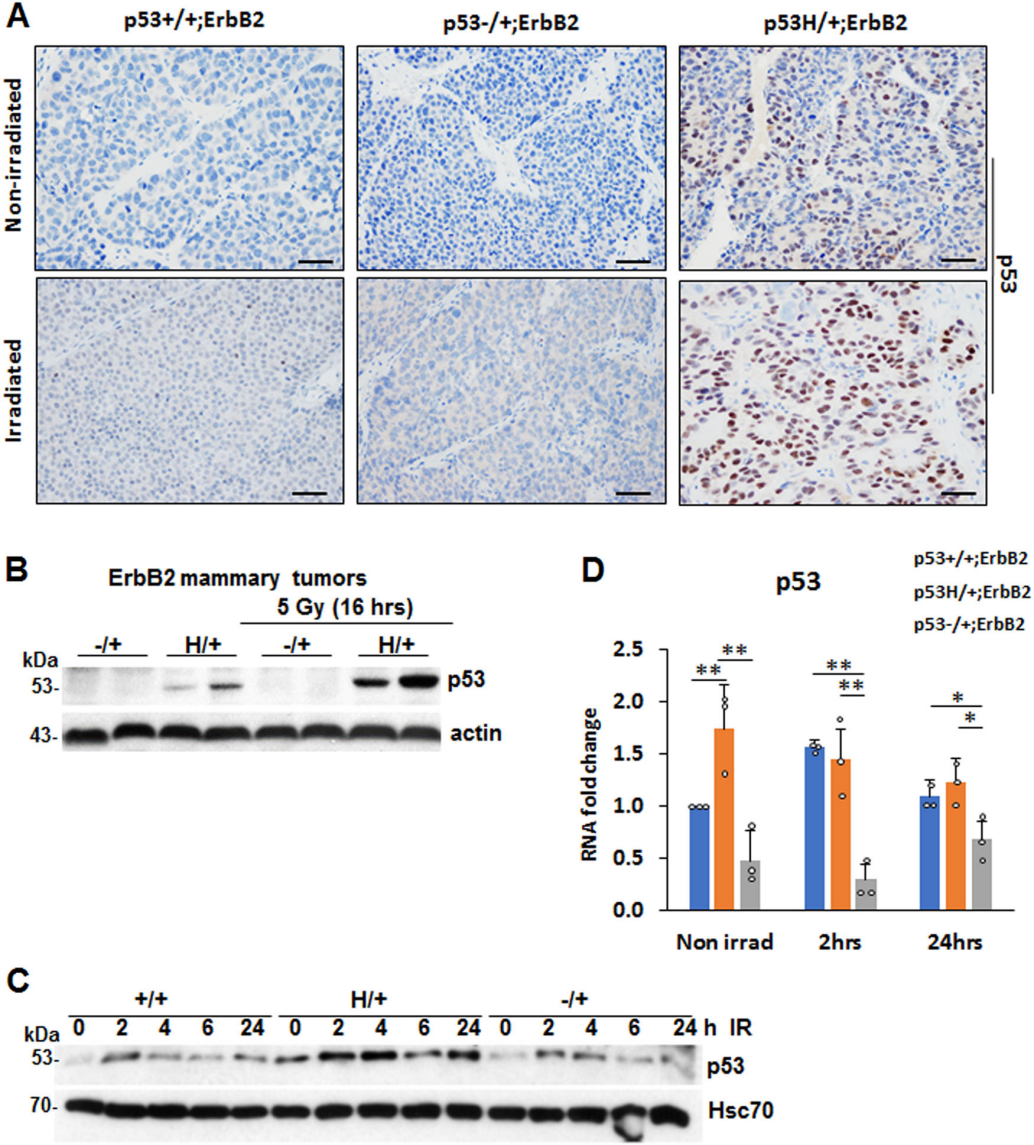
Irradiation induces the accumulation of mutant p53 protein in heterozygous cancer cells. **a** The increase in p53LOH in p53H/+;ErbB2 mammary tumors are associated with the stabilization of mutp53 after irradiation of pre-malignant lesions, while irradiation does not affect wtp53 levels in p53+/+;ErbB2 and p53−/+;ErbB2 tumors. Representative images of p53 IHC of mammary tumors with indicated genotypes that were non-irradiated and irradiated. Four tumors per genotype were analyzed. The scale bar represents 50 μm. **b** Irradiation stabilizes mutp53 protein in mutp53 heterozygous tumors, but not in p53−/+;ErbB2 tumors. Western blot 16 h after irradiation in vivo. Actin is a loading control. **c** wtp53 in p53+/+;ErbB2 cells was only transiently upregulated at 2 h post-irradiation (9 Gy), mutp53 shows much higher and continuous stabilization in p53H/+;ErbB2 cells. HSC70 is a loading control. **d** Irradiation in vitro does not induce p53 mRNA in p53H/+;ErbB2 cells, 24 h post-irradiation. *n* = 3 independent experiments. *p < 0.05; **p < 0.01; ***p < 0.001. Error bars represent ± SD.

Most homozygous mutp53 human cancers and cell lines accumulate high levels of mutp53 protein; however, little is known about how mutp53 protein levels are regulated in heterozygosity. Consistent with our previous study on R248Q;MMTV-Neu mouse model^11^, we found only 10–15% of p53 positive cells in p53H/+;ErbB2 tumors, while p53 staining was undetected in p53−/+;ErbB2 and p53+/+;ErbB2 tumors (Fig. 3a and Supplementary Fig. 2b). Irradiation-mediated p53LOH in p53H/+;ErbB2 mammary tumors was associated with significant stabilization of mutp53 protein in vivo (Fig. 3a, and Supplementary Fig. 2b) and in cell lines generated from mammary tumors that underwent p53LOH in vivo (Fig. 2d, lanes 9–11 and Supplementary Fig. 2a). Conversely, irradiation did not affect wtp53 levels in p53+/+;ErbB2 and p53−/+;ErbB2 tumors (Fig. 3a and Supplementary Fig. 2b). These results are consistent with Li et al. report that irradiation stabilizes mutp53 protein in MDA231 cells and, thus, promotes proliferation^14^. As mutp53 protein stabilization in tumors was proposed to be essential for its oncogenic function^15^, p53LOH with subsequent mutp53 stabilization may represent a key event in cancer progression in vivo.

Western blot of mouse tumors 16 h post-irradiation revealed that in irradiated p53H/+;ErbB2 tumors mutp53 protein was stabilized to a higher level than non-irradiated p53H/+;ErbB2 tumors, while p53 in p53−/+;ErbB2 tumors remained undetectable (Fig. 3b). Likewise, wtp53 in p53+/+;ErbB2 cell line was only transiently upregulated 2 h post-irradiation, but mutp53 showed much higher level in p53H/+;ErbB2 cell line up to 24 h after irradiation (Fig. 3c and Supplementary Fig. 2c).

It was previously shown that mutp53 mRNA is upregulated in response to genotoxic anthracyclines in human cell lines^16^. Analysis of p53 mRNA level showed no increase in p53 mRNA in p53H/+;ErbB2 cells after irradiation (Fig. 3d), suggesting post-transcriptional regulation of mutp53 protein levels in heterozygosity post-irradiation. Hence, we hypothesized that, in heterozygous cells, irradiation stabilizes mutp53 over the threshold that is sufficient to promote its oncogenic activities leading to p53LOH and tumor progression.

### P53LOH is associated with the switch from HRR to NHEJ and genomic instability

Genomic instability, such as chromosomal rearrangement caused mainly by failure in normal chromosome segregation during mitosis, has been regarded as one of the major causes of LOH in cancer^17,18^. Mutations in a number of genes, e.g., p53 and PI3K, hinder normal mitosis leading to chromosomal aberrations^17^. Alternatively, the accumulation of various oncogenic mutations during cancer progression can be a result of inefficient DNA repair. Therefore, we assessed two major DNA repair mechanisms in ErbB2 mammary tumors with various p53 genotypes.

Wtp53 is activated in response to genotoxic treatments, eliciting cell-cycle arrest, DNA repair, and/or apoptosis^19,20^. Depending on cell context and the extent of DNA-damage, p53 may promote DNA repair by one or both of the two major repair pathways: (1) homologous recombinational repair (HRR)^21,22^, and (2) nonhomologous end-joining (NHEJ)^22–24^. HRR is relatively slow and less error-prone, while NHEJ is faster and more error-prone^25^.

**Fig. 4:**
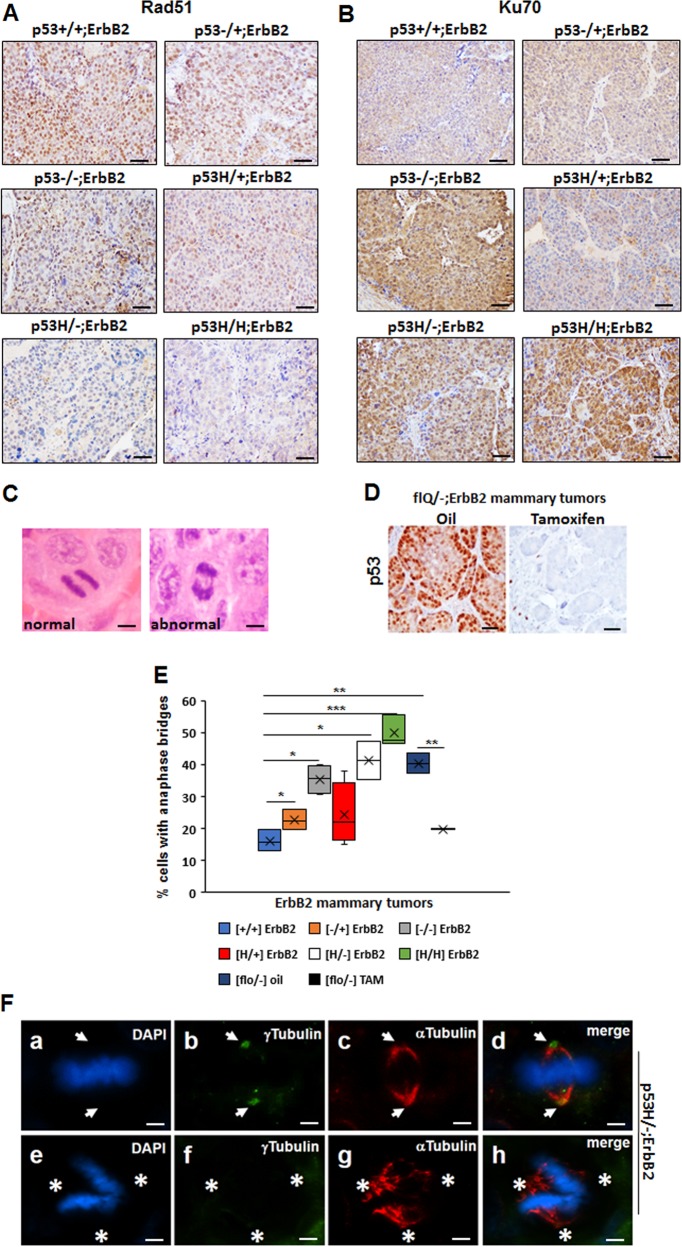
P53LOH is associated with the switch from homologous recombinational repair (HRR) to nonhomologous end joining (NHEJ) and genomic instability. **a** Rad51(marker for HRR) IHC in ErbB2 mammary tumors of mice with indicated p53 genotypes. **b** Ku70 (a marker for NHEJ) in ErbB2 mammary tumors of mice with indicated p53 genotypes. Four mammary tumors per genotype were stained. **c** H&E staining of normal anaphase showing the segregating masses of chromosomes and bridging (arrow) between the segregating masses of chromosomes during anaphase. **d** p53 IHC staining in the tumor from flR248Q/−;ErbB2 mouse injected with oil or following the depletion of p53 in the tumor from flR248Q/-;ErbB2 mouse after tamoxifen injection. The scale bars in A–D represent 50 μm. Images in A–D are representative stainings from 10 mice per group. **e** Quantification of anaphase bridges (AB) in ErbB2 mammary tumors of mice with indicated p53 genotypes. *n* = 3 tumors per genotype. *p < 0.05; **p < 0.01; ***p < 0.001. **f** Staining for mitotic spindles in a mitotic cell (metaphase) in p53H/-;ErbB2 mouse mammary tumor (**a**, **e**). Nuclear staining (DAPI), (**b**, **f**) centrosomes (γ-Tubulin), (**c**, **g**) mitotic spindles (α-Tubulin), (**d**, **h**) merge. **a**–**d** a mitotic cell with normal (2) spindle poles and 2 centrosomes. **e**–**h** a mitotic cell with no centrosomes (acentrosomal) and abnormal (>2) spindle poles. Arrows point to the position of the centrosomes in the mitotic cell. Asterisks indicate the three directions of the pull of the acentrosomal spindle poles. Data are representative of 10 images from 4 mice per genotype. The scale bar represents 145 μm.

HRR (Rad51 as a marker) was activated in p53+/+;ErbB2, p53−/+;ErbB2, p53H/+;ErbB2 and p53−/−;ErbB2 but was suppressed in p53H/−;ErbB2 and p53H/H;ErbB2 mammary tumors (Fig. 4a and Supplementary Fig. 3a). Conversely, wtp53 inhibited NHEJ (Ku70 as a marker), while higher Ku70 staining was only in tumors lacking wtp53 (Fig. 4b and Supplementary Fig. 3b). Hence, we hypothesized that, in the context of p53 status, the presence of wtp53 allele may shift DNA repair mechanism towards to HRR, whereas loss of wtp53 allele (LOH) leads to switch to NHEJ repair with mutp53 actively suppressing HRR, and causing the acquisition of multiple mutations, mitotic abnormalities, and chromosomal aberrations.

Chromosomal aberrations can be measured by the frequency of ‘anaphase bridges’ (AB) in the anaphase of the cell-cycle. AB are extended chromosome bridging between two spindle poles (Fig. 4c) and are a histologic hallmark of dicentric chromosomes^26^. High AB was shown to be associated with the increased frequency of Apc LOH in a colon cancer mouse model^18^. We found a marginal difference in AB scoring between p53+/+;ErbB2, p53−/+;ErbB2 and p53H/+;ErbB2 mammary tumors, whereas the absence of wtp53 allele markedly increased AB in ErbB2 mammary tumors (Fig. 4c). Additionally, p53H/−;ErbB2 tumors had higher AB compared to p53−/−;ErbB2 tumors and AB was further increased in p53H/H;ErbB2 tumors (Fig. 4e). Also, we analyzed another ErbB2 mouse model with conditional-deletion of R248Q mutp53 allele (flQ/−;ErbB2) upon tamoxifen administration^15^. Genetic ablation of R248Qp53 in vivo significantly reduced the mutp53 expression in established ErbB2 tumors compared to vehicle-treated tumors (Fig. 4d) and was concomitant with a two-fold AB decrease (Fig. 4e). Thus, our results indicate elevated AB independently of the type of p53 mutation compared to p53−/− tumors (Fig. 4e).

Several studies have implicated centrosome abnormalities and mitotic multipolar spindle formation, as the origin of chromosome instability in a variety of human tumors^27–30^. P53 is required for proper centrosome duplication and was shown to localize to the centrosomes^31–34^. To identify centrosome aberrations (>2 or absence of centrosomes), we analyzed mitotic cells in mammary tumors for centrosome and spindle formation. Indeed, we observed acentrosomal multipolar polar spindles in p53H/−;ErbB2 tumors (Fig. 4f) only.

Collectively our data suggest that in heterozygosity wtp53 enables the maintenance of the genomic integrity in cancer cells. It is plausible that DNA damage via stabilization of mutp53 protein shifts the balance between mutant and wtp53 alleles and unveils the oncogenic power of mutp53, leading to increased genomic aberrations and p53LOH. Consequently, loss of wtp53 allele leads to further genome perturbations fueling tumor progression.

### P53LOH is associated with the activation of the mTOR pathway

**Fig. 5:**
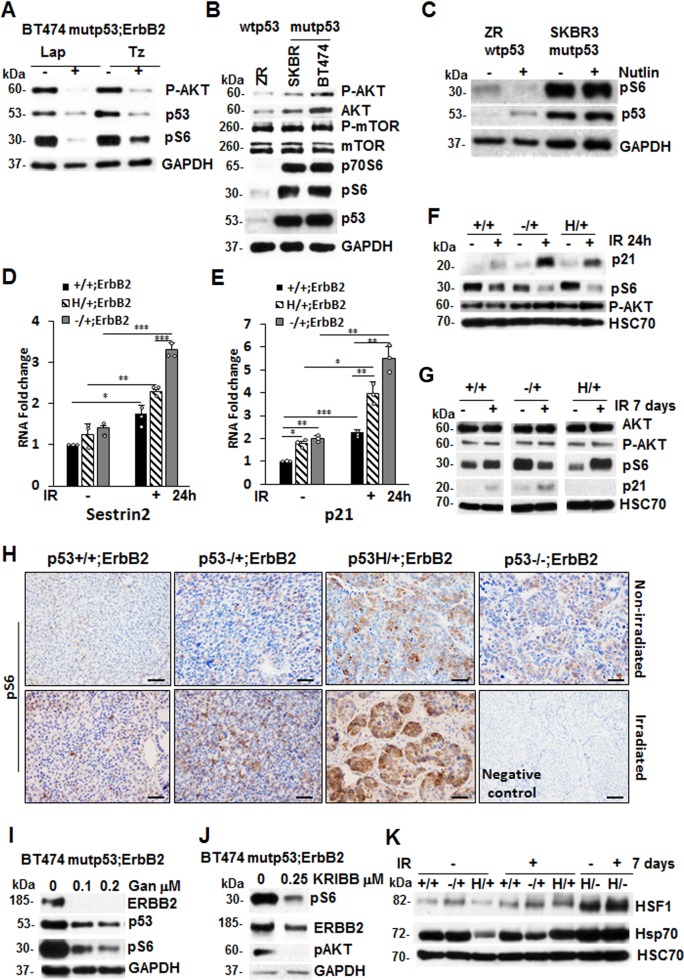
P53LOH is associated with the activation of the mTOR pathway. **a** ErbB2 inhibition by lapatinib and trastuzumab inhibits mTOR (pS6) in human mutp53 (BT474) cells. **b** The mTOR (pS6) pathway is more activated in mutp53;ErbB2 (BT474 and SKBR3) than in wtp53 cells (ZR75–30). **c** Upregulation of wtp53 by nutlin suppresses mTOR signaling in wtp53;ErbB2 cells ZR 75–30, but not in mutp53;ErbB2 SKBR3 cells. **d**, **e** Irradiation induces RNA expression of p53 targets Sestrin 2 (**d**) and p21 (**e**) in all genotypes p53+/+;ErbB2, p53−/+;ErbB2 and p53H/+;ErbB2 cells. QRT-PCR 24 h post-irradiation. *n* = 3 independent experiments. *p < 0.05; **p < 0.01; ***p < 0.001. **f** The mTOR (pS6) pathway is downregulated in the presence of wtp53 allele 24 h after irradiation that is concomitant with p21 upregulation. **g** irradiation-induced p53LOH in p53H/+;ErbB2 cells is associated with upregulation of mTOR and lack of detectable p21 in the long term. This is in contrast to p53+/+;ErbB2 and p53−/+;ErbB2 cells. Western blot 7 days post-irradiation. HSC70 as a loading control. **h** Irradiation-induced p53LOH is concomitant with the upregulation of mTOR signaling that is more profound in mutp53 heterozygous tumors. The scale bar represents 50 μm. Hsp90 inhibition by ganetespib (**i**) and HSF1 inhibition by KRIBB11 (**j**) suppresses mTOR in mutp53 human BT474 cells. Western blot after 24 h treatment with indicated concentrations. GAPDH as a loading control. **k** p53LOH after irradiation is associated with both mTOR and HSF1 activation (as indicated by elevated Hsp70) only in p53H/+;ErbB2 cells. Western blot 7 days after irradiation. HSC70 as a loading control. Error bars represent ± SD. Experiments were repeated three times with similar results.

The mTOR pathway is a key downstream component of ErbB2 signaling^35^. Indeed, specific inhibitors of ErbB2 (lapatinib and trastuzumab) effectively suppressed mTOR, as indicated by downregulation of pS6, a downstream target of mTOR, (Fig. 5a and Supplementary Figs. 4a and 5a). The mTOR pathway plays an essential role in regulating many oncogenic processes – such as genomic instability in different cancer types^18,36,37^, including breast cancer^36,38^. The stimulation of the mTOR pathway followed by translational deregulation and accelerated G1-S transition was implicated in inducing genomic instability and Apc LOH in a colon cancer mouse model^18^. Hence, we asked whether the increased genomic instability and elevated p53LOH observed in the presence of mutp53 (Fig. 2, Table 1) is attributed to increased mTOR signaling.

Several studies showed that wtp53 inhibits the mTOR pathway via inducing Sestrin 1 and 2 expressions, that interact and activate AMPK leading to mTOR inhibition^39,40^. Our data show elevated mTOR signaling in mutp53;ErbB2 vs. wtp53;ErbB2 human cancer cells as indicated by high levels of downstream effectors of mTOR—p70S6 and pS6, whereas the level of mTOR and p-mTOR protein were comparable (Fig. 5b and Supplementary Figs. 4b and 5b). Furthermore, upregulation of wtp53 by nutlin suppressed mTOR signaling in wtp53;ErbB2 cells, but not in mutp53;ErbB2 cells (Fig. 5c and Supplementary Figs. 4c and 5c). Consistent with transcriptional activity of wtp53 (Fig. 2f), Sestrin 2 and p21 mRNA expression was upregulated 24 h post-irradiation in all mouse cell lines genotypes (Fig. 5d–e), and this upregulation was associated with downregulation of mTOR activity (Fig. 5f and Supplementary Fig. 5d). Importantly, irradiation did not alter pAKT, the upstream effector of mTOR, (Fig. 5f and Supplementary Fig. 5d), indicating that wtp53-mediated induction of Sestrins is the main regulator of mTOR activity post-irradiation.

To investigate the effect of p53LOH on mTOR activity, we tested cells 7 days post-irradiation (Fig. 2f). Compared to p53+/+;ErbB2, the loss of wtp53 allele in p53H/+;ErbB2 cells was associated with mTOR upregulation and p21 suppression (Fig. 5g and Supplementary Fig. 5e), while there were sustained mTOR inhibition and p21 upregulation in p53−/+;ErbB2 cells (Fig. 5g). Similarly, irradiation in vivo exacerbated p53LOH concomitant with significant upregulation of mTOR signaling in p53H/+;ErbB2 tumors (Fig. 5h and Supplementary Fig. 6a, b; Table 1).

Next, we asked whether mutp53 impacts the mTOR pathway through a gain-of-function (GOF) mechanism. We previously showed that mutp53 amplifies ErbB2 signaling via stimulation of HSF1 and its transcriptional target Hsp90, which, in turn, stabilizes numerous Hsp90 clients, such as ErbB2 and mutp53 itself^41^. The mTOR pathway components, which are Hsp90 clients (https://www.picard.ch/downloads/Hsp90interactors.pdf), may also be stabilized by mutp53-HSF1-Hsp90 loop. Indeed, inhibition of both Hsp90 and HSF1, efficiently suppressed mTOR signaling in mutp53;ErbB2 cell lines BT474 (Fig. 5I, j and Supplementary Fig. 6c, d) and SKBR3 (Supplementary Fig. 4a–c). Furthermore, p53LOH post-irradiation was associated with the activation of both mTOR and HSF1 (as indicated by its elevated target, Hsp70) only in p53H/+;ErbB2 cells (Fig. 5k and Supplementary Fig. 6e). Hence, in addition to the loss of wtp53 suppressive activity, p53LOH may lead to mTOR activation via stimulation of HSF1-ErbB2 axis in a mutp53-dependent manner, providing the survival advantage over p53+/+;ErbB2 and p53−/+;ErbB2 cells. Thus, the activation of the mTOR pathway associated with p53LOH may generate selective pressure for the loss of wtp53 allele in p53H/+;ErbB2 cells.

### Cells with mutant p53 have defective DNA-damage repair response and cell-cycle profile following γ-irradiation

Upon genotoxic stress, wtp53 activates the transcription of genes involved in cell-cycle arrest and DNA repair or apoptosis, to protect the genome from the accumulation of mutations, while mutp53 may perturb these genome-guarding mechanisms and promote genomic instability^19,42^. Yet, how p53 heterozygous cells respond to DNA damage is not fully understood.

**Fig. 6:**
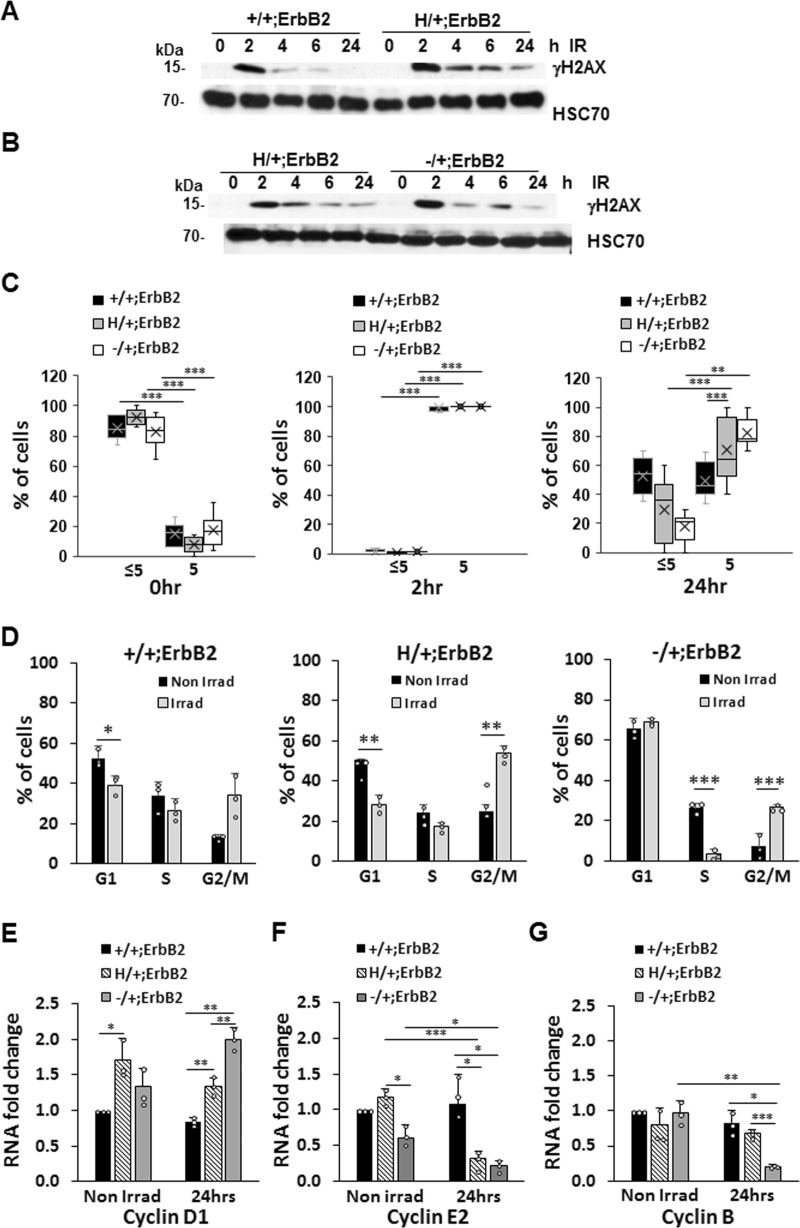
Cells with mutant p53 have defect both in DNA-damage repair response and in cell-cycle profile following γ-irradiation (**a**, **b**) Western blot of γH2AX level (representing DNA damage) post-irradiation (9 Gy, single dose) showing γH2AX efficient resolution in p53+/+;ErbB2 cells (**a**) but is sustained up to 24 h in p53H/+;ErbB2 and p53−/+;ErbB2 cells (**b**). HSC70 as a loading control. **c** Quantification of cells with >5 and <5 γH2AX foci/cell in p53+/+;ErbB2, p53H/+;ErbB2, and p53−/+;ErbB2 cell lines, before and after γ-irradiation (9 Gy, 2 and 24 h post-irradiation). **d** Aberrant cell-cycle checkpoint following γ-irradiation in p53H/+;ErbB2 cells. Bar graphs showing cell-cycle analysis of p53+/+;ErbB2, p53H/+;ErbB2, and p53−/+;ErbB2 cell lines irradiated (gray bars) or not (black bars). **e**, **f** QRT-PCR 24 h post-irradiation is showing the impact of a single dose of γ-irradiation (9 Gy) on the transcription of Cyclin D1 (**e**), Cyclin E2 (**f**), Cyclin B (**g**). Level of transcripts was quantified relatively to HPRT. *n* = 3 independent experiments. *p < 0.05; **p < 0.01; ***p < 0.001. Error bars represent ± SD.

Hence, we irradiated (9 Gy) cultured mouse mammary tumor cells and examined the extent of DNA damage using γH2AX as a marker of DNA double-strand breaks. Western blot analysis (Fig. 6a, b and Supplementary Fig. 7) and foci assessment (Fig. 6c) showed sustained DNA damage up to 24 h in p53H/+;ErbB2 cells. Conversely, in p53+/+;ErbB2 cells, γH2AX peaked at 2 hr post-irradiation, was efficiently resolved by 4 h, and resumed to a normal level by 24 h post-irradiation (Fig. 6a, c and Supplementary Fig. 7). These results suggest that, while p53+/+ cells exhibit a functional DNA-damage response, wtp53 haploinsufficient cells manifest persistent DNA damage due to a deficient DNA repair following γ-irradiation.

Next, we compared cell-cycle profiles of cells with various genotypes 24 h after γ-irradiation. Non-irradiated p53+/+;ErbB2 and p53−/+;ErbB2 cells exhibited comparable cell-cycle profiles, whereas p53H/+;ErbB2 cells showed cell-cycle profile with lower G1 and S and significantly higher G2/M indicating an increased rate of proliferation (Fig. 6d). Consistent with fast recovery from DNA-damage post-irradiation, p53+/+;ErbB2 cells did not significantly change G1 and S content and had a slight increase in G2/M arrest (Fig. 6d). In p53−/+;ErbB2 cells, irradiation-induced G1 and G2/M arrest, and significantly reduced S-phase (Fig. 6d). Conversely, p53H/+;ErbB2 cells continued cell cycling with sustained DNA damage (Fig. 6a, b), as indicated by the unchanged S-phase and increased G2/M (Fig. 6d).

We then evaluated the expression of cyclin D1, cyclin E, and cyclin B 24 h post-irradiation. Cyclin D1 is essential for G1-S progression^43–45^, and its level varies by cell-cycle phase^43–45^. At basal level all three cell lines had similar cyclin D1 transcription (Fig. 6e), though p53H/+;ErbB2 cells tended to have a higher level (1.5-fold higher than p53+/+;ErbB2) reflecting a higher overall proliferation. Consistent with unchanged S-phase post-irradiation, cyclin D1 transcription level remained unchanged in both p53+/+;ErbB2 and p53H/+;ErbB2 cell as compared to non-irradiated controls (Fig. 6e). However, p53−/+;ErbB2 cells showed the highest increase in cyclin D1 transcription post-irradiation (Fig. 6d) consistent with G1 arrest and diminished S-phase.

Cyclin E prepares cells for DNA replication during the G1-S transition and is required for centrosome duplication in the S-phase^46^. While there was no significant difference in cyclin E2 transcription level in non-irradiated cell lines, both p53H/ + ;ErbB2 and p53−/+;ErbB2 cells showed a significant reduction in cyclin E2 transcription post-irradiation. This result suggests that following DNA-damage wtp53 induces growth arrest in p53−/+;ErbB2 cells, while this mechanism malfunctions in p53H/+;ErbB2 cells, which enter S-phase unprepared for correct centrosome number and DNA duplication. Additionally, our results indicate that following DNA-damage cyclin E requires both wtp53 alleles, whereas p53 haploinsufficiency leads to inadequate cyclin E expression (Fig. 6f).

Cyclin B is required for mitotic spindle assembly and entry into mitosis^47^. There was no significant difference in cyclin B transcription level in non-irradiated cell lines (Fig. 6g). However, irradiation induced a significant cyclin B transcription reduction only in p53−/+;ErbB2 cells, indicating a blockage in entering mitosis. In agreement with elevated G2/M-phase post-irradiation, cyclin B showed a marginal increase in p53H/+;ErbB2 cells indicating a transition to mitosis with unrepaired DNA.

Hence, in p53H/+;ErbB2 cells, the aberrant G1-S transition coupled with defective DNA repair may generate the genomic plasticity that facilitates p53LOH. In turn, p53LOH upregulates the mTOR pathway to further enhance cancer cells fitness and enable their survival after DNA damage.

## Discussion

As the predictive value of mutp53 status in response to genotoxic therapies remains controversial^5^, here we analyzed the oncogenic impact of mutant R172H p53 on the development and progression of mammary tumors after irradiation. Previously we showed that in heterozygosity mutant p53 R172H is a more potent activator of ErbB2-mediated mammary tumorigenesis than simple loss of p53^12^.

In the current study, we demonstrated that a single dose of irradiation, at the time of onset of pre-malignant lesions, profoundly accelerated mammary tumorigenesis in heterozygous, but not in p53+/+;ErbB2 mice (Fig. 2a, b). We identified a novel oncogenic activity of mutp53 where it exacerbated p53LOH in response to irradiation, which correlated with enhanced metastasis only in the presence of mutp53 allele. To our knowledge this has not been reported before. Despite the difference in p53LOH rate, irradiation equally shortened the survival of p53H/+;ErbB2 and p53−/+;ErbB2 mice (Fig. 2a). This could be attributed to an alternative p53LOH-independent mechanism(s) in tumors in response to irradiation. In addition, mouse cancer models have limitations in recapitulating human disease, e.g. the survival analysis of mice reflects only the rate of tumor growth since mice were usually sacrificed when the tumor reaches a certain size. Conversely, 90% of deaths of breast cancer patients are a consequence of metastasis. In relation to human data, we observed in p53H/+;ErbB2 mice a strong association between enhanced p53LOH and the increased rate of metastases (Table 1). Also, we previously noted significant stabilization of mutp53 protein in metastatic lesions as opposed to heterogeneous p53 staining in primary p53H/+;ErbB2 mammary tumors^12^, which is consistent with the p53LOH phenotype (Fig. 3a and Supplementary Fig. 2b)^11^. We speculate that mutp53 stabilization after p53LOH, with the subsequent induction of genomic instability (Fig. 4), may lead to the acquisition of metastatic properties in cancer cells.

In support of our in vivo findings, the analysis of METABRIC human database demonstrated stage-dependent benefit from genotoxic modalities for patients with mutp53 breast cancer: improved survival of stage 2 patients, but the shorter survival of stage 1 patients (Fig. 1a–h) that strongly correlates with p53LOH status (80 and 48% respectively) (Fig. 1i). Conversely, patients with wtp53 tumors, benefit from radiotherapy independently of the stage. In support of our hypothesis, a previous study showed that TP53/KRAS co-mutations are predictive of the deleterious effect of adjuvant chemotherapy compared to KRAS mutation alone^6^. Hence, studies are needed to determine whether negative outcomes from genotoxic therapies in the early stages of mutp53 breast cancer patients are caused by radiation-induced p53LOH.

Our data on survival of non-irradiated mice (Fig. 2a, Table 1,^12^) implies, that despite the transcriptional activity of wtp53 towards a subset of targets (Figs. 2f and 5d–e) mutp53 may exert DN function in heterozygous mammary tumors. Notably, neither straight p53H/+ knock-in mice^48,49^ nor the H allele crossed into the Ras, EμMyc^50^ or Wnt^10^ models demonstrated shortened survival compared to their p53null counterparts, suggesting that mutp53 contributes to tumorigenesis only in cooperation with particular oncogenic drivers, such as ErbB2. This is supported by clinical data showing that TP53 mutations are associated with poor prognosis in HER2-positive breast cancer patients, but not in patients with luminal A and basal-like tumors despite the high frequency of TP53 mutations^51^.

Furthermore, oncogenic activities of mutp53 in heterozygosity were manifested by the increased rate of p53LOH and metastasis post-irradiation (Table 1). These outcomes were associated with the continuous stabilization of mutp53 protein post-irradiation in vivo and in vitro, while wtp53 upregulation was quickly resolved after stress (Fig. 3b, c). It is conceivable that in heterozygosity the ratio of wtp53 to mutp53 defines the oncogenic function of mutp53, either through DN effect or GOF, and irradiation can drive mutp53 protein level over a threshold necessary to manifest its oncogenic activity. In support, heterozygous expression of mutp53 (R270H) exerted DN effect on tumor latency, multiplicity, and progression only after UV exposure but not spontaneous tumors^52^. In R246S mutp53 knock-in mouse model, the DN effect on transactivation was detectable only after acute p53 activation^53^. Also, DNA damage increased mutp53 DN activity in various tissues of p53H/+; ErbB2 mice^48,49^.

Nevertheless, we were unable to clearly detect DN effect of mutp53 on the analyzed subset of wtp53 targets post-irradiation, as p21 and sestrin 2 mRNA was upregulated in heterozygous cells 24 h post-irradiation (Fig. 5d–e). These results are consistent with previous report on the MMTV-Wnt1 mouse model showing that wtp53 induces reversible p21-mediated growth arrest in p53H/+;ErbB2 tumors after doxorubicin treatment^10^. Our data on the differential expression of mTOR pathway and p21 post-irradiation (Figs. 2e, 5g–h) indicates that p53LOH may be the key oncogenic event that overrides irradiation-induced growth arrest and inhibition of metabolic activity.

Wtp53 is crucial in regulating key cellular processes such as proliferation and the maintenance of genomic integrity^19,20^. Conversely, mutp53 is an important driver of genomic instability,^19,54^ which may constitute the main mechanism underlying p53LOH after irradiation. Our data show differential deployment of HRR vs. NHEJ DNA repair mechanisms depending on p53 genotype where the presence of wtp53 allele correlated with HRR, while after p53LOH mutp53 may inhibit HRR and induce NHEJ (Fig. 4), thus enhancing genomic instability. This is consistent with a previous study showing that mutp53 allows the bypassing of G2/M DNA-damage checkpoint, causing inefficient HRR in a lymphoma mouse model^42^. Additionally, the frequency of AB formation correlated with the lack of wtp53 allele, whereas mutp53 exacerbated AB in the absence of wtp53 (Fig. 4c, e) and correlated with aberrant centrosome formation and multipolar spindles (Fig. 4f).

Furthermore, our data suggest that DNA-damage hinders the wtp53 genome-protective function, leading to persistent DNA damage in both p53H/+;ErbB2 and p53−/+;ErbB2 cells. Conversely, DNA damage was quickly resolved in p53+/+;ErbB2 cells (Fig. 6a–c). Importantly, persistent DNA damage-induced growth arrest in p53−/+;ErbB2 cells, whereas p53H/+;ErbB2 cells were able to overcome p21-mediated G1 arrest and enter mitosis with unrepaired DNA and defective centrosome duplication. In support, transcriptome analysis of TSGA database showed mutant TP53-associated dysregulation of cell-cycle regulatory genes in the majority of human cancer types, including breast cancer^55^.

It remains unclear how mutp53 heterozygous cells escape DNA-damage-induced growth arrest. One possibility is that p53 targets, other than p21 and Mdm2, are affected by stabilized mutp53, leading to the defective checkpoint and cell-cycle progression. Indeed, the expression of ~50% of genes induced by wtp53 was significantly altered in the presence of mutp53 in KRAS lung cancer model suggesting that DN effect of mutp53 might be selective towards the specific subset of genes^56^. Alternatively, stabilized mutp53 in heterozygosity can mitigate wtp53-mediated suppression of the mTOR pathway after irradiation. As a result, the enhanced mTOR signaling may accelerate the G1-S transition in p53H/+;ErbB2 cells with erroneously repaired DNA, leading to p53LOH. In support, previous studies showed the importance of the mTOR pathway in regulating every phase of the cell-cycle progression, partly by interacting with cyclin E and cyclin B [reviewed in ref. ^57^]. Also, the stimulation of the mTOR pathway, followed by G1-S acceleration was implicated in genomic instability and Apc LOH in a colon cancer mouse model^18^.

How can mutp53 promote mTOR signaling? Earlier work by us and others showed that, depending on the oncogenic environment, mutp53 could drive tumorigenesis by activating a number of growth factor receptors implicated in the activation of PI3K/Akt signaling^58–61^, including ErbB2^12^, that are upstream of the mTOR pathway. Previously we demonstrated that mutp53 enhances ErbB2 signaling via HSF1-Hsp90 axis^12^. Additionally, components of the mTOR pathway, as a Hsp90 clients (https://www.picard.ch/downloads/Hsp90interactors.pdf), may also be stabilized by mutp53-HSF1-Hsp90 cascade. Indeed, ErbB2, HSF1, and Hsp90 inhibition suppress mTOR signaling (Fig. 5a, i–k). Thus, mutp53 may contribute to mTOR activation by stimulating HSF1-ErbB2 axis, and/or by direct interaction and suppression of AMPK signaling^62^.

**Fig. 7:**
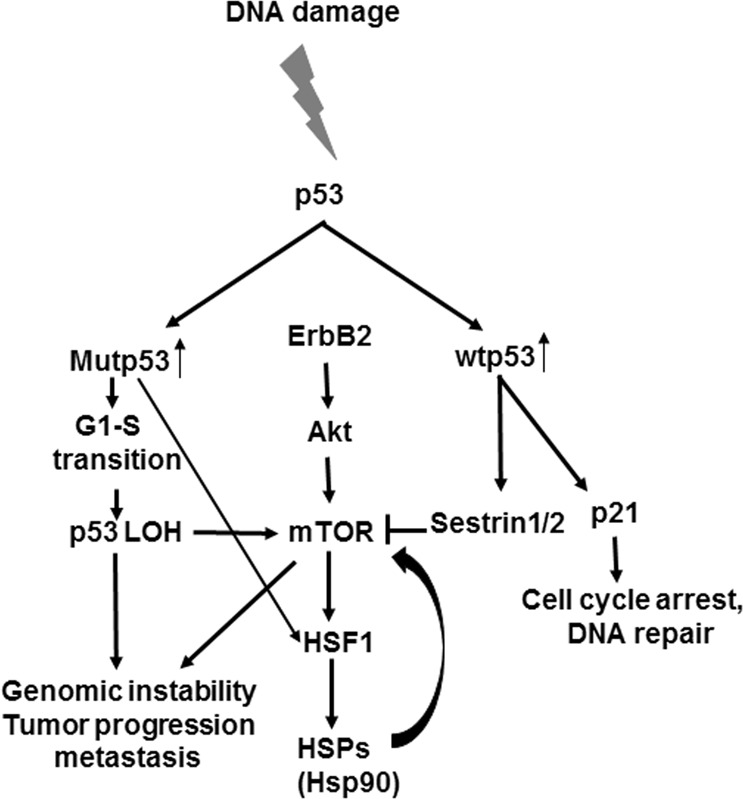
Molecular mechanisms of p53 loss of heterozygosity in breast cancer in response to DNA damage. Proposed model for the role of mutp53 in promoting tumorigenesis. Following DNA damage, p53 expression is induced. Wtp53 would induce cell-cycle arrest and suppression of the mTOR pathway. On the other hand, mutp53 would promote cell-cycle progression with unrepaired DNA, LOH and cell survival via activation of the mTOR pathway through HSF1-HS90 axis, eventually leading to genomic instability, tumor progression, and metastasis.

Collectively, our data suggest a two-phase response to irradiation-induced DNA damage where mutp53 may play a role in promoting LOH (Fig. 7): acute response to irradiation (phase 1) and the recovery phase (phase 2). In phase 1, p53+/+;ErbB2 cells activate efficient DNA-damage repair, and after its completion resume cell cycling. In p53−/+;ErbB2 cells, irradiation induces sustained cell-cycle arrest due to deficient DNA-damage repair (high p21, low cyclin E and B1, and suppressed S-phase). In p53H/+;ErbB2 cells, mutp53 interferes with the regulation of the cell-cycle checkpoint to induce cell-cycle arrest despite inefficient DNA repair (high p21, low cyclin E, high cyclin B1, and increased G1-S transition and G2/M).

In phase 2, p53+/+;ErbB2 cells resume normal cell cycling (low p21 and close-to-normal mTOR) after DNA-damage repair. Conversely, in p53−/+;ErbB2 cells unrepaired DNA induces persistent cell-cycle checkpoint and the suppression of cell-cycle (high p21 and low mTOR). In p53H/+;ErbB2 cells, mutp53 via enhanced mTOR signaling (and possibly other mechanisms) forces the cell to by-pass cell-cycle checkpoint (p21 lacking, high level of mTOR), despite DNA is not efficiently repaired. Consequently, aberrant cell-cycle progression with damaged DNA leads to p53LOH followed by enhancement of genomic instability (NHEJ DNA repair, centrosome abnormalities) and loss of p21-mediated checkpoints enabling unrestricted proliferation. In support, it was previously demonstrated that loss of wtp53-mediated p21 expression induces the transcription of hTERT after irradiation, confering proliferation and radio-resistance in HER2-positive breast cancer^63^.

In the current work, we mainly studied R175H p53 mutation, which was identified as a hotspot in ErbB2 breast cancer^64^. Our study on mutant p53 R248Q/+;Neu mouse breast cancer model demonstrated that loss of wtp53 allele is required for mutp53 stabilization^11^ and GOF activities, promoting chromosomal aberration. This suggests that our conclusions can be applied to a variety of p53 mutations. Still, it remains undetermined whether irradiation accelerates p53LOH and tumorigenesis in the presence of various types of p53 mutations, and in other types of cancers.

In sum, our study may have significant implications on the therapeutic interventions for early stages of human breast cancer and help to prevent the potentially deleterious effects of genotoxic therapies and conquer the problem of overtreatment in breast cancer.

## Methods

### Metabric data

Human Metabric data analysis, of the somatic mutation profiles of 2433 breast cancers, was done using data from a retrospective study(10). The data are deposited and is publicly available at http://www.cbioportal.org. The analysis was done using the program and tools made available online at http://www.cbioportal.org.

### Mice

MMTV-ErbB2 mice carrying activated ErbB2 (strain FVBN-Tg(MMTV-ErbB2)NK1Mul/J) were from Jackson Labs. p53 R172H (called p53H/H) and control p53 null (p53−/−) mice (C57Bl6J background) were a gift from G. Lozano^49^. p53H/−;ErbB2 mice were generated by crossing ErbB2 mice with p53−/− mice and then breeding the p53+/−;ErbB2 progeny with p53H/H mice. p53H/-;ErbB2 mice were then crossed to generate p53H/H;ErbB2 and p53−/−;ErbB2 females for analysis. p53+/+;ErbB2 were generated from crossing of p53H/+;ErbB2 and p53+/−;ErbB2 mice. Mice carrying the floxed p53R248Q mutation (referred to as floxQ) was generated as described before^15^. For all mice genotypes, only female littermates were used for all analyses. Animals were monitored weekly to determine their breast cancer and sarcoma onset and were promptly killed when their tumors reached 4 cm^3^ in volume or when animals appeared moribund. Careful necropsies were performed, and tumors and all major organs collected, fixed in 10% formalin, embedded in paraffin and sectioned for histopathologic analysis. For survival analysis, P-values were determined by log-rank analysis. Mice were treated according to guidelines approved by the Institutional Animal Care and Use Committee at Stony Brook University.

### Cell lines

Human ErbB2-positive breast cancer cell lines ZR-75–30 carrying wild-type TP53, and BT474, SKBR3, carrying E285K, R175HTP53 mutations, respectively, were purchased from ATCC. Where shown, cells were treated with indicated concentrations of HER2 inhibitors lapatinib (L-4899, LC Lab) and Trastuzumab (gift of Dr. A. Kudelka), ganetespib (HSP90 inhibitor) (STA-9090, Synta Pharmaceuticals), or KRIBB11 (HSF1 inhibitor) (385570, Calbiochem). Establishing mouse mammary tumors cell lines was described before^65^. Mouse mammary tumor cell lines: p53+/+;ErbB2, p53H/+;ErbB2 and p53+/−;ErbB2, were isolated from their corresponding mammary tumors and maintained in culture. Mouse mammary epithelial cells (MECs) p53H/H;ErbB2 and p53−/+;ErbB2 were also isolated and maintained in culture. Isolated mouse mammary tumor cell line clones 176.3 p53+/+;ErbB2, 136.12 p53+/+;ErbB2, 134.9 p53H/+;ErbB2 and 221 p53H/−;ErbB2 were selected for all cell culture experiments.

### Gamma irradiation

Mice were exposed to total-body γ-irradiation with a 137Cs source, with a dose rate of 0.8 Gy/min, for a total of 5 Gy. Another group of mice (sham) were placed in the room without being exposed to irradiation. Animals were either observed for survival post-irradiation or were killed by CO_2_ asphyxiation followed by cervical dislocation at set times after irradiation, and the mammary tumors were removed for further analysis. For the survival experiment, all animals (irradiated or not) were monitored weekly to determine their breast cancer and sarcoma onset and were promptly killed when their tumors reached 3 cm^3^ in volume or when animals appeared moribund. Necropsy, tumor removal, and fixation and analysis were carried out as described above. For γ-irradiation of cells, a total of 9 Gy was used. Non-irradiated cells (sham) were placed in the room without being exposed to irradiation.

### Immunofluorescence and immunohistochemistry

H&E staining was performed by the Research Histology Core Laboratory, Department of Pathology, Stony Brook University. For tissue immunostaining, sections were deparaffinized in xylene, rehydrated in ethanol gradient, and then treated with 10 mM Na citrate buffer, pH 6.0, at 120 °C for 10 min in a pressure cooker. The histological sections were incubated with a blocking buffer [10% normal horse serum (NHS) and 0.01% Tween 20 in 1x Tris-buffered PBS (TTBS)] for 1 h at 37 °C. Sections were then stained using rabbit anti-p53 (1:200; Santa Cruz Biotechnology), rabbit anti-pS6 (1:100; Abcam), mouse anti-γTubulin (1:100 clone D10 Santa Cruz Biotechnology), rabbit anti-αTubulin (1:500 cell signal), rabbit anti-Rad51 (1:100; Abcam), and rabbit anti-Ku70 (1:100; Abcam), at 4 °C overnight. Washes were done using TTBS, and detection of primary antibodies for immunofluorescence (IF) was carried out using appropriate Alexa Fluor-labeled secondary antibodies (Molecular Probes) at 1:500 dilutions for 30 min at 37 °C, counterstained with Hoechst 33258 (2 μg/ml), mounted with Prolong gold (Molecular Probes), and cover-slipped. For IHC, secondary unconjugated bovine anti-rabbit antibody (Jackson Immuno Research), were added at 1:300 dilution in 10% NHS in TTBS for 30 min at 37 °C. After being washed, goat anti-bovine horseradish peroxidase-conjugated tertiary antibody was used at 1:500 dilution 30 min at 37 °C. All antibody dilutions were made in 10% NHS in TTBS. The color was developed using substrate-chromogen solution, counterstained with hematoxylin, and then mounted. For IF on cells, media aspired from cells grown on chamber slides, cells were fixed with methanol at −20 °C for 10 min, and then washed 3X with PBS. Cells were incubated with blocking buffer [10% normal horse serum (NHS) and 0.01% Tween 20 in PBS], for 1 h at 37 °C. Cells were then stained with rabbit anti-γH2AX (1:200, Cell Signaling) for 1 h at 37 °C, and then washed 3X with PBS. Goat anti-rabbit Alexa fluor-labeled 568 secondary antibodies (Molecular Probes) at 1:500 dilution for 30 min at 37 °C, counterstained with Hoechst 33258 (2 μg/ml), mounted with Prolong gold (Molecular Probes), and cover-slipped. Images for H&E and IHC slides were acquired at ×400 total magnification using Olympus microscope (Olympus) equipped with Olympus DP72 camera. For IF, images were acquired at x600 total magnification using a Nikon Eclipse Ti-S microscope (Nikon Instruments) equipped with QI-Click camera (QImaging). Where applicable, quantification of IHC staining intensity was performed, using ImageJ^66^, on 10 images of randomly selected fields per genotype per treatment. For γH2AX foci, cells were counted from at least five randomly selected fields.

### Anaphase bridging index (ABI)

The ABI was determined as described before^18^. A minimum of 50 anaphases per mouse (3–5 per group) was scored from H&E sections.

### Determination of LOH of the *p53+* locus

DNA was extracted from frozen mouse mammary tumors, using QIAmp DNA Micro Kit (Qiagen). An equal amount of DNA was used for PCR amplification of p53 locus using primers described before^49^. For loading control, we used primers for ROSA locus: [F], 5’-AAAGTCGCTCTGAGTTGTTAT-3’, [R], 5’-TAAGCCTGCCCAGAAGACTC-3’. An equal volume of the amplified product was electrophoresed through a 1.5% agarose gel. Amplified DNA bands were visualized, and the image captured using FluoroChem HD2 (ProteinSimple). LOH was determined based on the presence or absence of the amplified wild-type band.

### Real-time PCR

All real-time PCR was done on cultured cells using 3 biological replicas. For determining p53 wild-type and R172H allele copy number in cultured cells, DNA was extracted from cultured cells using QIAmp DNA Micro Kit (Qiagen). An equal amount of DNA was used for PCR amplification of p53 locus using the following primers: p53 wild-type [F], 5’-CACATGACGGAGGTCGTGAGTTG-3’, R172H mutation [F], 5’-CACATGACGGAGGTCGTGAGTTA-3’, [R], 5’-CTGTCTTCCAGATACTCGGGATAC-3’. The primers were designed to detect the nucleotide point mutation G→A that results in amino acid mutation R172H. The specificity of the primers was validated by DNA extracted from p53+/+;ErbB2, p53H/+;ErbB2, p53−/+;ErbB2, p53−/−;ErbB2 and p53H/H;ErbB2 mouse tails. Genotypes of generated cell lines were confirmed by quantitative real-time PCR (qPCR).

For determination mRNA transcript level, RNA was extracted from cultured cells using Trizol as per manufacturer instructions. For cDNA synthesis, 200 ng/sample was used in a 20 μl reaction volume prepared from QuantiTect Reverse Transcription Kit (Qiagen). After cDNA synthesis, the reaction volume was diluted to 200 μl using DEPC-treated water. For qPCR, 1 μl of the diluted cDNA was used per reaction volume. The following primers were used: allele-specific p53 wild-type [F], 5’-CACATGACGGAGGTCGTGAGTTG-3’, R172H mutation [F], 5’-CACATGACGGAGGTCGTGAGTTA-3’, [R], 5’-CTGTCTTCCAGATACTCGGGATAC-3’; total p53 [F], 5’-CACATGACGGAGGTCGTGAGAC-3’, [R], 5’-CTGTCTTCCAGATACTCGGGATAC-3’; p21 [F], 5’-CCTGGTGATGTCCGACCTG-3’, [R], CCATGAGCGCATCGCAATC-3’; sestrin2 [F], 5’-ACACCCGGACTACCTTAGCA, [R], 5’-TGGGAACCCACCAGGTAAGA-3’; cyclin D1 [F], 5’-GCGTACCCTGACACCAATCTC-3’, [R], 5’-CTCCTCTTCGCACTTCTGCTC-3’; cyclin E2 [F], 5’-ATGTCAAGACGCAGCCGTTTA-3’, [R], 5’-GCTGATTCCTCCAGACAGTACA-3’; cyclin B [F], 5’-AAGGTGCCTGTGTGTGAACC-3’, [R], 5’-GTCAGCCCCATCATCTGCG-3’. For loading control, we used Hprt primers^67^: [F], 5’- GGCTATAAGTTCTTTGCTGACC-3’, [R], 5’- CTCCACCAATAACTTTTATGTCC-3’. For all real-time PCR, amplification was done using Quantitech sybr green (Qiagen) reaction mixture, and detection was done using QuantStudio3 (Thermo Fisher Scientific).

### Cell-cycle analysis

Cultured cells were harvested by trypsinization and pelleted by spinning at 1500 r.p.m. for 10 min. The cells pellet was twice with PBS, and the cells pellet fixed in 70% ethanol. The cells were pipetted gently up and down to loosen the cells in a suspension and stored in −20 °C overnight. The cells were then pelleted by spinning at 1500 r.p.m. for 10 min, washed once in PBS, then resuspended in permeabilization buffer (0.25% tritonX100 in PBS) and incubated for 15 min at RT. The cells were then pelleted and resuspended in staining solution (20 μg/ml propidium iodide and 10 μg/ml RNase A in PBS), and incubated in the dark on ice for 30 min before analysis. Cell-cycle analysis by flow cytometry was done at Stony Brook Flow-cytometry Core Facility, using Becton Dickinson FACSCAN analyzer.

### Immunoblot analysis

For immunoblots, cell lysates with equal total protein content (2–20 μg) were blotted with antibodies to p53 (FL393), p21 GAPDH, Hsc70 (all from Santa Cruz Biotechnology); ErbB2, AKT, pAKT, p-mTOR, mTOR, p70S6, pS6, γH2AX (all from Cell Signaling); HSF1 Hsp70, (all from Enzo Life Sciences Inc.).

### Statistics and reproducibility

All statistical analysis between groups was done using the *t*-test. Significance was determined at *p* < 0.05. All immunoblots were repeated at least two times.Cell culture experiments were repeated three times.

### Reporting summary

Further information on research design is available in the Nature Research Reporting Summary linked to this article.

## Supplementary information

Supplementary Information

Description of Additional Supplementary Files

Supplementary Data 1

Reporting Summary

## Data Availability

The Human Metabric data set used here is available at http://www.cbioportal.org. Source data can be found in Supplementary Data 1. All other data are available from authors on reasonable request.
