# Supplementary material for: Irradiation induces p53 loss of heterozygosity in breast cancer expressing mutant p53
Source: Commun Biol. 2019 Nov 27;2:436. doi: 10.1038/s42003-019-0669-y (PMC6881331; doi:10.1038/s42003-019-0669-y)
Supplement: Supplementary file 1 — Supplementary Information [file 42003_2019_669_MOESM1_ESM.pdf]

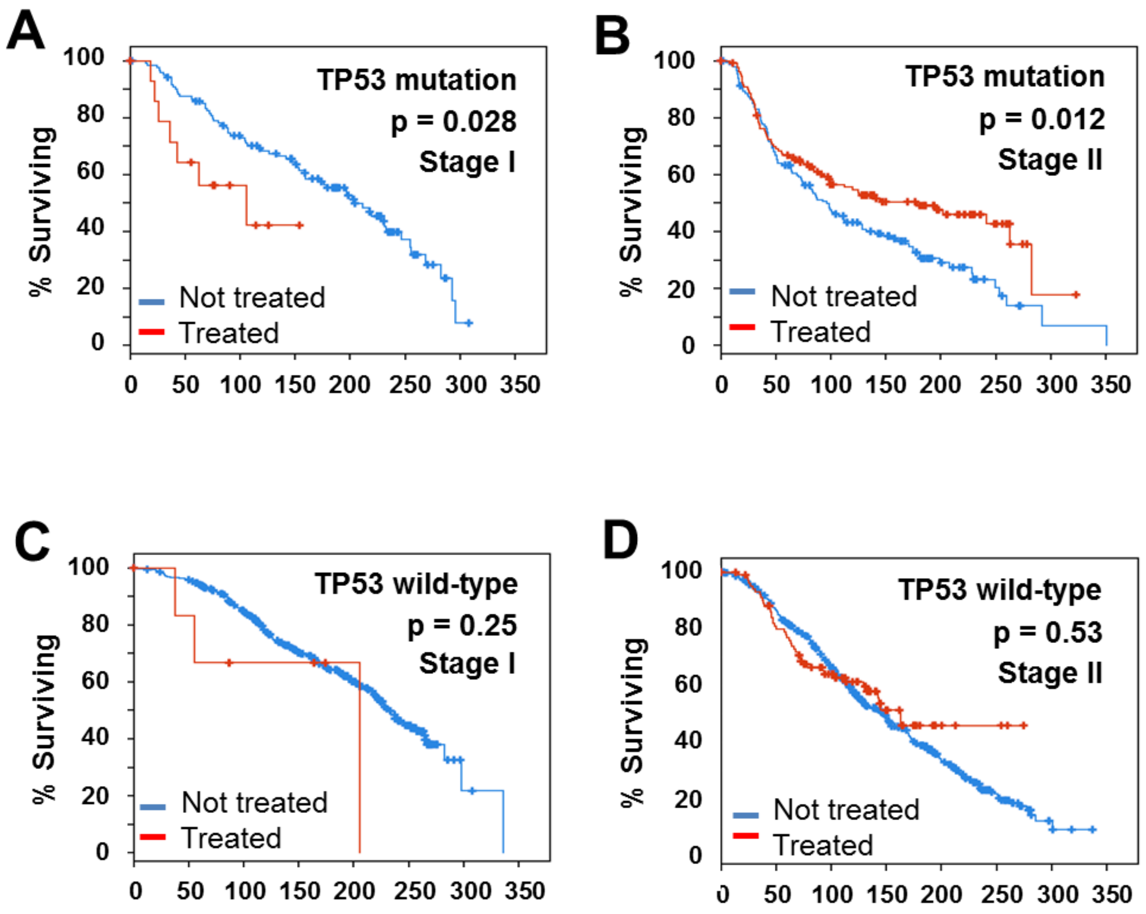

Supplementary Figure 1. The survival of mutp53 breast cancer patients following chemotherapy is stage dependent.

Kaplan-Meier survival curve for patients with breast cancer receiving chemotherapy (red line) or untreated (blue line) according to the stage and TP53 mutation status. **(A)** p53 mutant, stage I; **(B)** p53 mutant, stage II; **(C)** wtp53 stage I; **(D)** wtp53 stage II.

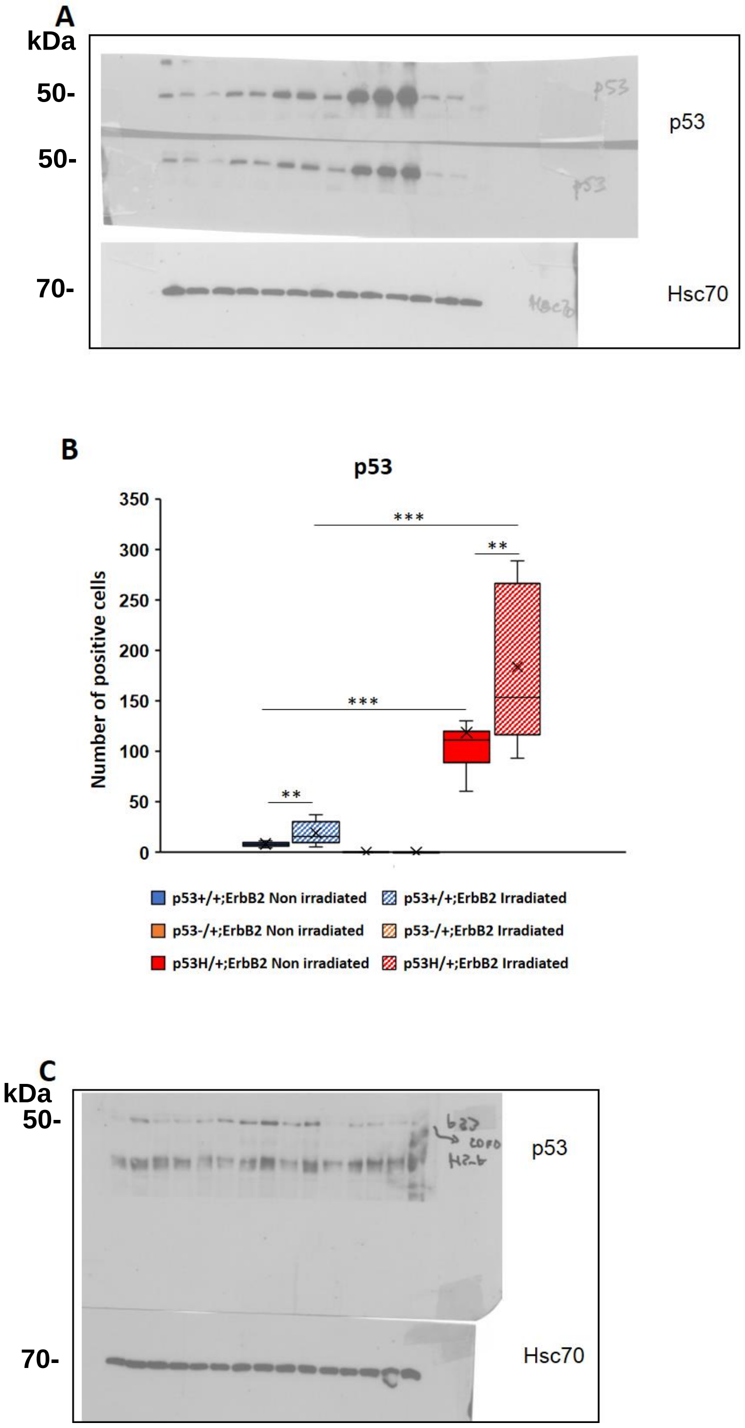

Supplementary Figure 2. Quantification of IHC staining and western blots shown in full.

(A) Western blot from Fig. 2D. **(B)** Quantification of the number of p53-positive cells in ErbB2 mouse mammary tumors. **(C)** Western blot from Fig. 3C. *=p≤0.05; **=p≤0.01; ***=p<0.001, ±SE.

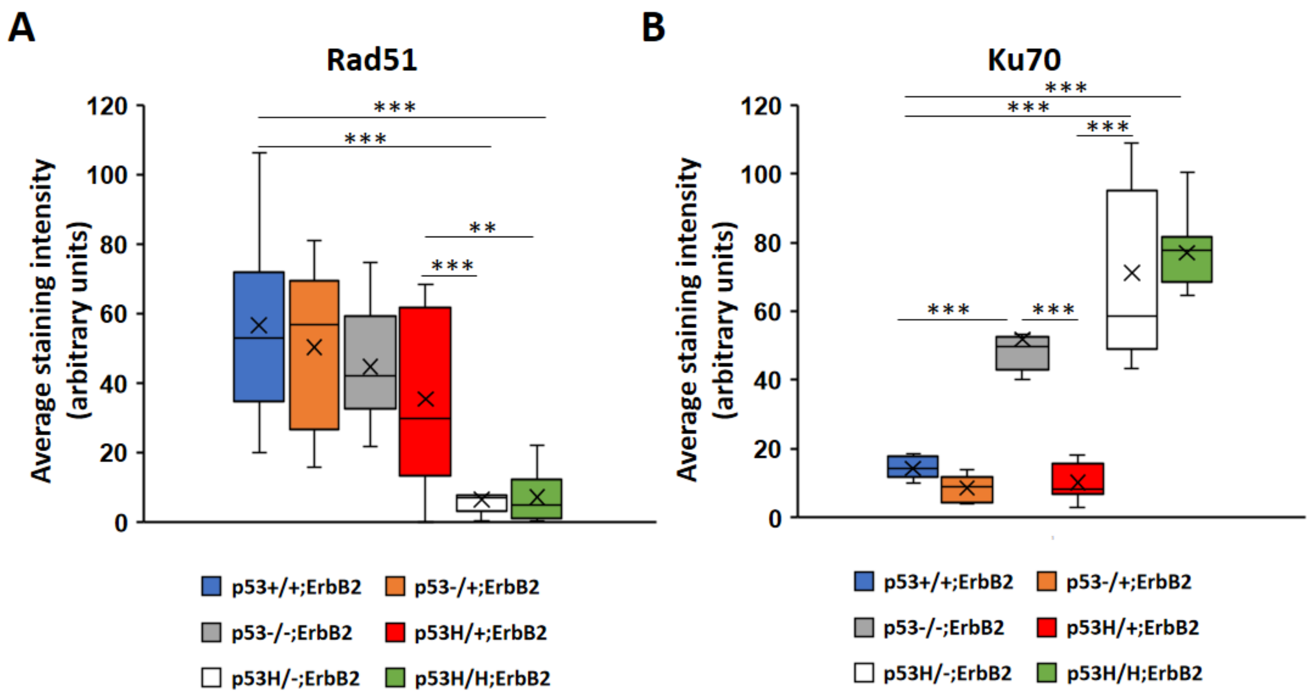

Supplementary Figure 3. Quantification of IHC staining.

(A) Quantification of staining intensity of Rad51 in ErbB2 mouse mammary tumors. **(B)** Quantification of staining intensity of Ku70 in ErbB2 mouse mammary tumors. *=p≤0.05; **=p≤0.01; ***=p<0.001, ±SE.

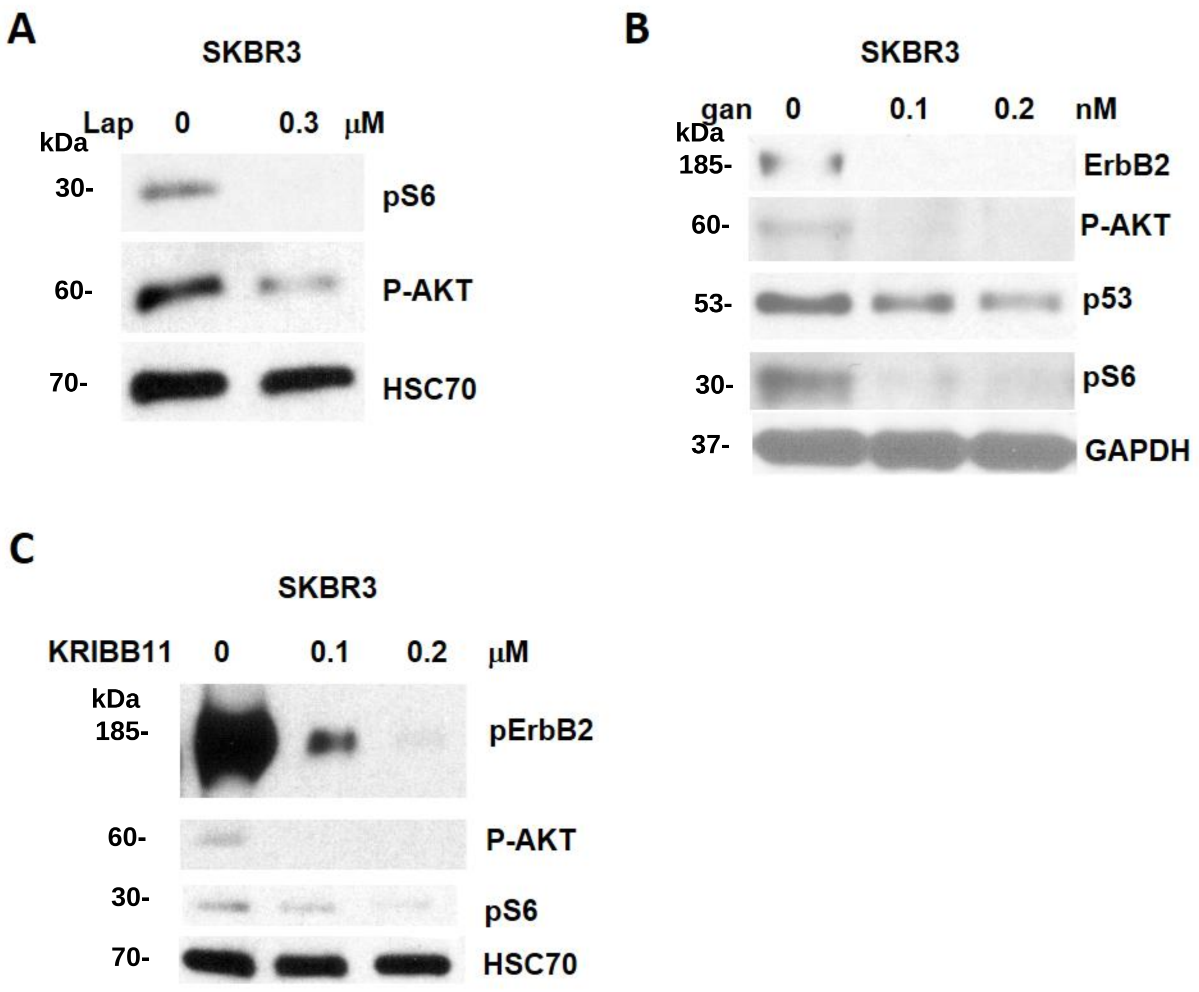

Supplementary Figure 4. Inhibition of mTOR pathway in SKBR3 cell line.

(A) ErbB2 inhibition by lapatinib inhibits mTOR (pS6) in human mutp53 (SKBR3) cells. Hsp90 inhibition by ganetespib **(B)** and HSF1 inhibition by KRIBB11 **(C)** suppresses mTOR in mutp53 human SKBR3 cells. Western blot after 24h treatment with indicated concentrations. HSC70 or GAPDH as a loading control.

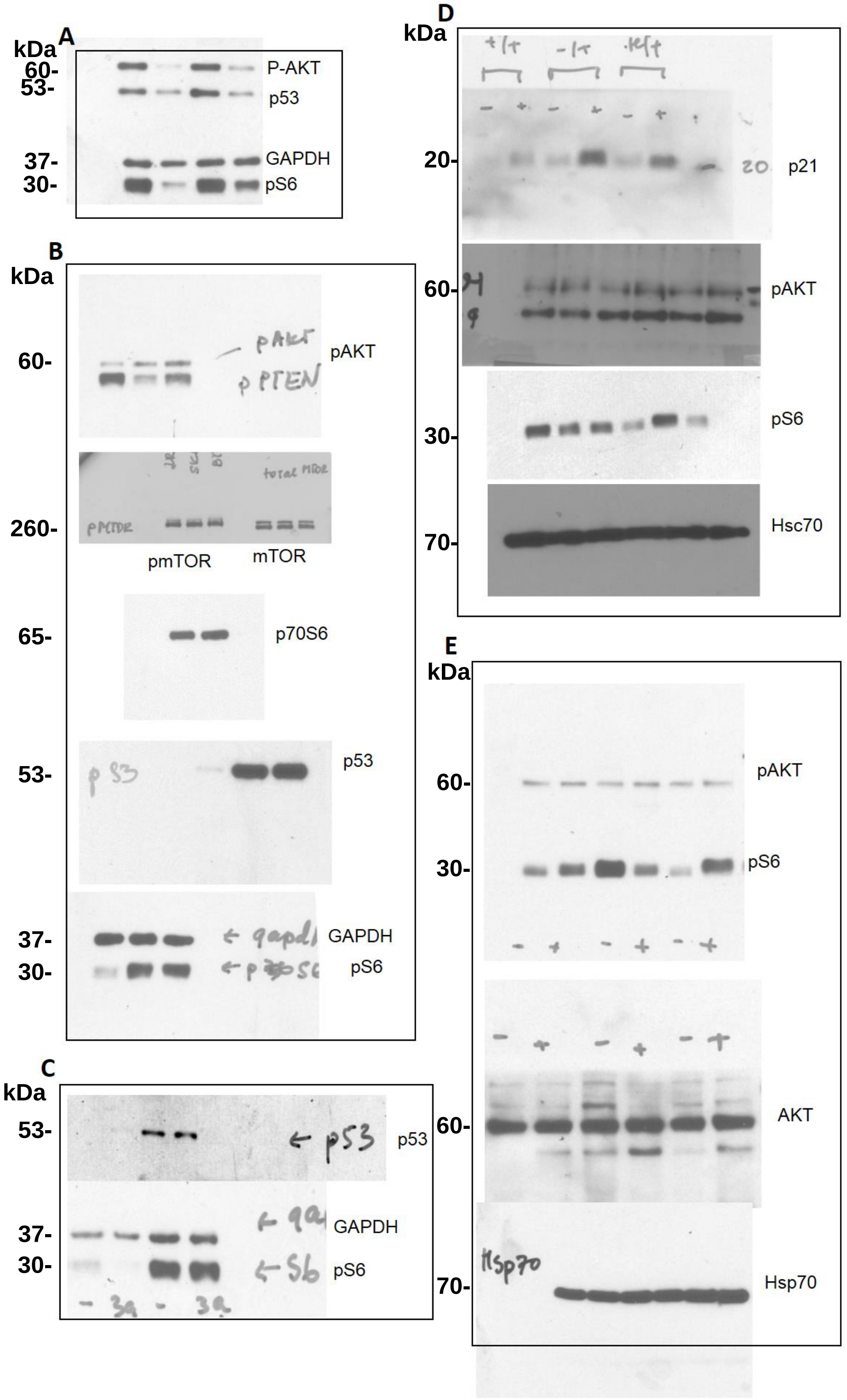

Supplementary Figure 5. Western blots shown in full.

(A) Western blot from Fig. 5A. **(B)** Western blot from Fig. 5B. **(C)** Western blot from Fig. 5C. **(D)** Western blot from Fig. 5F. **(E)** Western blot from Fig. 5G.

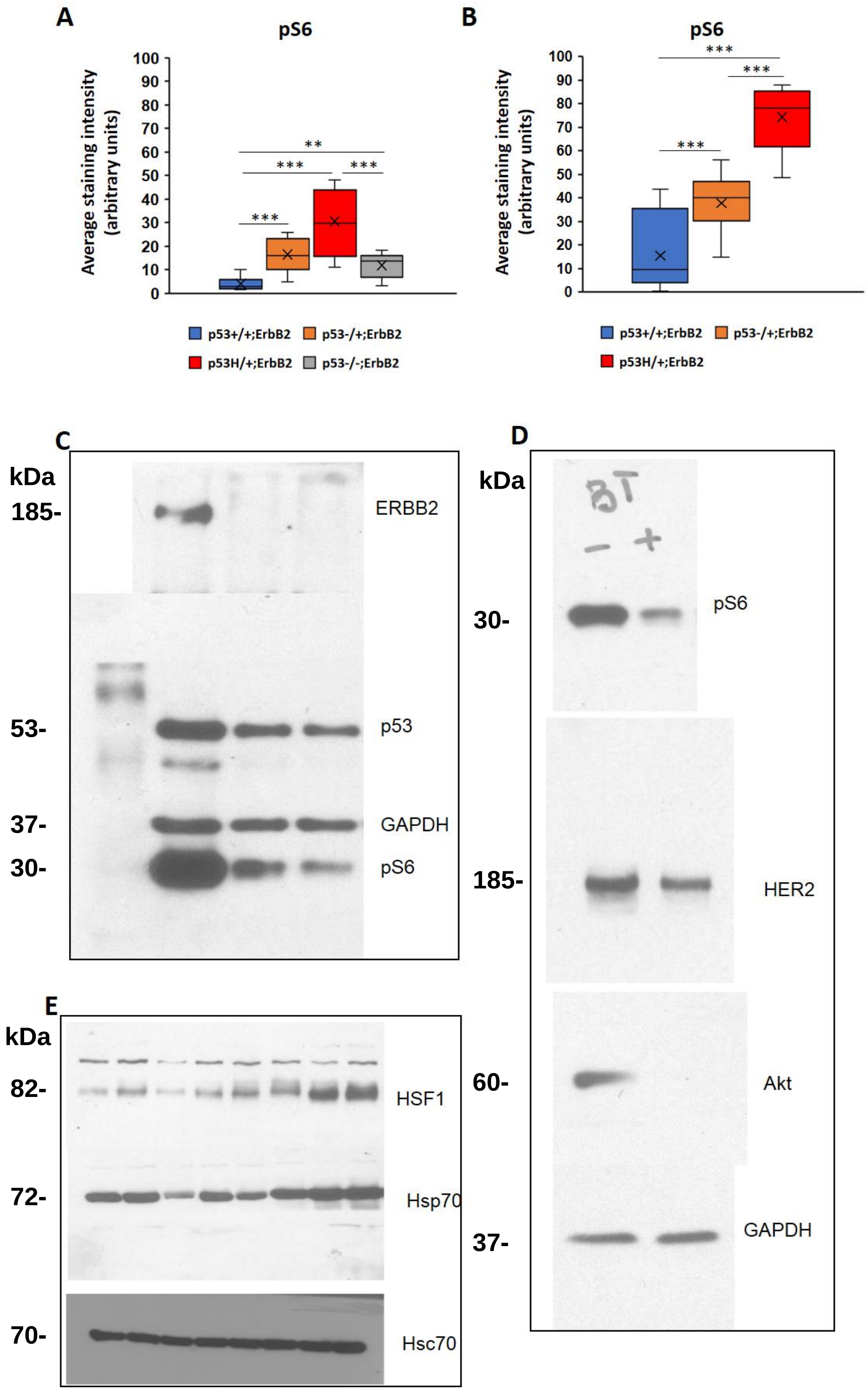

Supplementary Figure 6. Quantification of IHC staining and western blots shown in full.

(A) Quantification of staining intensity of pS6 in non-irradiated ErbB2 mouse mammary tumors. **(B)** Quantification of staining intensity of pS6 in irradiated ErbB2 mouse mammary tumors. **(C)** Western blot from Fig. 5I. **(D)** Western blot from Fig. 5J. **(E)** Western blot from Fig. 5K. *=p≤0.05; **=p≤0.01; ***=p<0.001, ±SE.

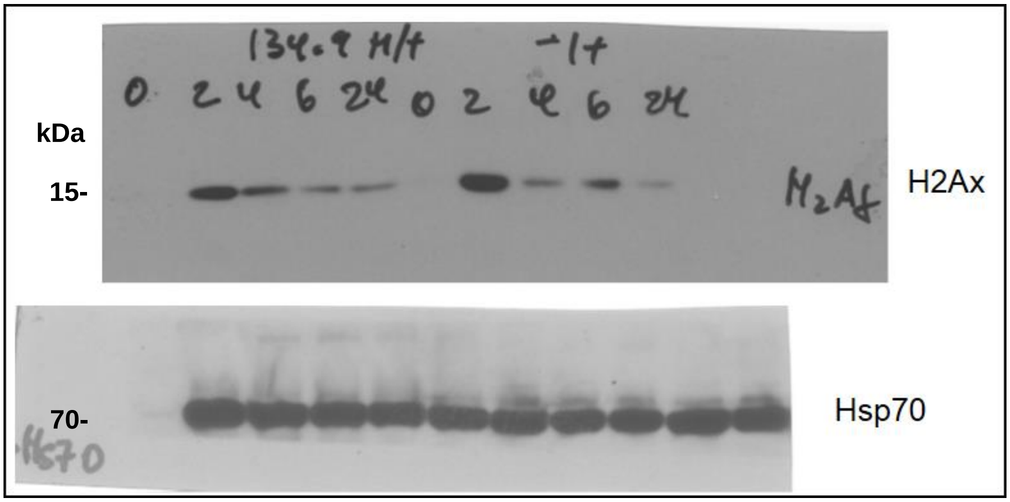

Supplementary Figure 7. Western blots shown in full.

(A) Western blot from Fig. 6B.
