# Supplementary material for: Irradiation induces p53 loss of heterozygosity in breast cancer expressing mutant p53
Source: Commun Biol. 2019 Nov 27;2:436. doi: 10.1038/s42003-019-0669-y (PMC6881331; doi:10.1038/s42003-019-0669-y)
Supplement: Supplementary file 2 — Description of Additional Supplementary Files [file 42003_2019_669_MOESM2_ESM.pdf]

Supplementary data 1:

Data used for real time PCR quantification in Figures 2,3,5 and 6 and for anaphase-bridges quantification in Figure 4.
