# Supplementary material for: Irradiation induces p53 loss of heterozygosity in breast cancer expressing mutant p53
Source: Commun Biol. 2019 Nov 27;2:436. doi: 10.1038/s42003-019-0669-y (PMC6881331; doi:10.1038/s42003-019-0669-y)
Supplement: Supplementary file 4 — Reporting Summary [file 42003_2019_669_MOESM4_ESM.pdf]

nature research

Corresponding author(s): Natalia Marchenko

Last updated by author(s): Sep 22, 2019

Reporting Summary

Nature Research wishes to improve the reproducibility of the work that we publish. This form provides structure for consistency and transparency in reporting. For further information on Nature Research policies, see Authors & Referees and the Editorial Policy Checklist.

Statistics

For all statistical analyses, confirm that the following items are present in the figure legend, table legend, main text, or Methods section.

n/a	Confirmed	
☐	☒	The exact sample size (*n*) for each experimental group/condition, given as a discrete number and unit of measurement
☐	☒	A statement on whether measurements were taken from distinct samples or whether the same sample was measured repeatedly
☐	☒	The statistical test(s) used AND whether they are one- or two-sided<br>*Only common tests should be described solely by name; describe more complex techniques in the Methods section.*
☐	☒	A description of all covariates tested
☒	☐	A description of any assumptions or corrections, such as tests of normality and adjustment for multiple comparisons
☐	☒	A full description of the statistical parameters including central tendency (e.g. means) or other basic estimates (e.g. regression coefficient) AND variation (e.g. standard deviation) or associated estimates of uncertainty (e.g. confidence intervals)
☐	☒	For null hypothesis testing, the test statistic (e.g. *F*, *t*, *r*) with confidence intervals, effect sizes, degrees of freedom and *P* value noted<br>*Give P values as exact values whenever suitable.*
☒	☐	For Bayesian analysis, information on the choice of priors and Markov chain Monte Carlo settings
☒	☐	For hierarchical and complex designs, identification of the appropriate level for tests and full reporting of outcomes
☐	☒	Estimates of effect sizes (e.g. Cohen's *d*, Pearson's *r*), indicating how they were calculated

Our web collection on statistics for biologists contains articles on many of the points above.

Software and code

Policy information about availability of computer code

Data collection	Assessment of the predictive effect of TP53 mutations in response to irradiation we investigated publicly available databases of retrospective clinical data study of Metabric cohort (>2,433 breast cancer patients, http://www.cbioportal.org) [Jackson JG, Pant V, Li Q, Chang LL, Quintas-Cardama A, Garza D, et al. p53-mediated senescence impairs the apoptotic response to chemotherapy and clinical outcome in breast cancer. Cancer Cell 2012;21(6):793-806.]
Data analysis	Analysis of patient survival was done using the program and tools made available online at http://www.cbioportal.org.

For manuscripts utilizing custom algorithms or software that are central to the research but not yet described in published literature, software must be made available to editors/reviewers. We strongly encourage code deposition in a community repository (e.g. GitHub). See the Nature Research guidelines for submitting code & software for further information.

Data

Policy information about availability of data

All manuscripts must include a data availability statement. This statement should provide the following information, where applicable:

- Accession codes, unique identifiers, or web links for publicly available datasets
- A list of figures that have associated raw data
- A description of any restrictions on data availability

Databases used for retrospective clinical data study of Metabric cohort (>2,433 breast cancer patients) is available at http://www.cbioportal.org. No restrictions on data availability.

Field-specific reporting

Please select the one below that is the best fit for your research. If you are not sure, read the appropriate sections before making your selection.

☒ Life sciences ☐ Behavioural & social sciences ☐ Ecological, evolutionary & environmental sciences

For a reference copy of the document with all sections, see nature.com/documents/nr-reporting-summary-flat.pdf

Life sciences study design

All studies must disclose on these points even when the disclosure is negative.

Sample size	For mice studies, at least 9 mice per group per treatment was used
Data exclusions	No data were excluded.
Replication	For in vitro experiments, each was made with 3 biological replicas to ensure reproducibility. all attempts were successful.
Randomization	Allocation of mice to each experimental group was based solely of their genotype
Blinding	For mice data analysis and for IHC the person doing the analysis was blinded to the mouse genotype.

Reporting for specific materials, systems and methods

We require information from authors about some types of materials, experimental systems and methods used in many studies. Here, indicate whether each material, system or method listed is relevant to your study. If you are not sure if a list item applies to your research, read the appropriate section before selecting a response.

Materials & experimental systems

n/a	Involved in the study	
☐	☒	Antibodies
☐	☒	Eukaryotic cell lines
☒	☐	Palaeontology
☐	☒	Animals and other organisms
☒	☐	Human research participants
☒	☐	Clinical data

Methods

n/a	Involved in the study	
☒	☐	ChIP-seq
☒	☐	Flow cytometry
☒	☐	MRI-based neuroimaging

Antibodies

Antibodies used	rabbit anti-p53 (1:200; Santa Cruz Biotechnology), rabbit anti-pS6 (1:100; Abcam), mouse anti-gamma-Tubulin (1:100 clone D10 Santa Cruz Biotechnology), rabbit anti-alphaTubulin (1:500 cell signal), rabbit anti-Rad51 (1:100; Abcam), and rabbit anti-Ku70 (1:100; Abcam).<br>Immunofluorescence secondary antibodies were from molecular probes.<br>For western blot, antibodies to p53 (FL393), p21 GAPDH, Hsc70 (all from Santa Cruz Biotechnology); ErbB2, AKT, pAKT, p-mTOR, mTOR, p70S6, pS6, gamma-H2AX (all from Cell Signaling); HSF1 and Hsp70, (all from Enzo Life Sciences Inc.).
Validation	For anti-p53 antibodies, mouse tissues or cell lines that that express or are null for p53 were used for validation.

Eukaryotic cell lines

Policy information about cell lines

Cell line source(s)	Human ErbB2-positive breast cancer cell lines ZR-75-30 carrying wild type TP53, and BT474, SKBR, carrying E285K, R175HTP53 mutations respectively, were purchased from ATCC.<br>Mouse mammary tumor cell lines: p53+/+;ErbB2, p53H/+;ErbB2 and p53+/-;ErbB2, were isolated from their corresponding mammary tumors and established and maintained in culture.
Authentication	Mouse mammary tumor cell lines were authenticated by their expression of ErbB2 and by genotyping for p53 status.
Mycoplasma contamination	The cells used are negative for mycoplasma contamination.
Commonly misidentified lines (See ICLAC register)	No commonly misidentified cell lines were used in this study.

Animals and other organisms

Policy information about studies involving animals; ARRIVE guidelines recommended for reporting animal research

Laboratory animals	MMTV-ErbB2 mice carrying activated ErbB2 (strain FVBN-Tg(MMTV-ErbB2)NK1Mul/J) were from Jackson Labs. p53 R172H (called p53H/H) and control p53 null (p53-/-) mice (C57Bl6J background) were a gift from G. Lozano (48). p53H/-;ErbB2 mice were generated by crossing ErbB2 mice with p53-/- mice and then breeding the p53+/-;ErbB2 progeny with p53H/H mice. p53H/-;ErbB2 mice were then crossed to generate p53H/H;ErbB2 and p53-/-;ErbB2 females for analysis. p53+/+;ErbB2 were generated from crossing of p53H/+;ErbB2 and p53+/-;ErbB2 mice. Mice carrying the floxed p53R248Q mutation (referred to as floxQ) was generated as described before (14). For all mice genotypes, only female littermates were used for all analyses.
Wild animals	The study did not involve wild animals
Field-collected samples	The study did not involve field-collected samples
Ethics oversight	Mice were treated according to guidelines approved by the Institutional Animal Care and Use Committee

Note that full information on the approval of the study protocol must also be provided in the manuscript.
